# A WRKY transcription factor, TaWRKY42-B, facilitates initiation of leaf senescence by promoting jasmonic acid biosynthesis

**DOI:** 10.1186/s12870-020-02650-7

**Published:** 2020-09-29

**Authors:** Ming-Ming Zhao, Xiao-Wen Zhang, Yong-Wei Liu, Ke Li, Qi Tan, Shuo Zhou, Geng Wang, Chun-Jiang Zhou

**Affiliations:** 1 Ministry of Education Key Laboratory of Molecular and Cell Biology, Hebei Collaboration Innovation Center for Cell Signaling, College of Life Sciences, Hebei Normal University, Shijiazhuang, 050024 Hebei China; 2 Institute of Genetics and Physiology, Hebei Academy of Agriculture and Forestry Sciences /Plant Genetic Engineering Center of Hebei Province, Shijiazhuang, 050051 Hebei China

**Keywords:** Leaf senescence, WRKYs, JA, Transcriptional regulation, Wheat

## Abstract

**Background:**

Leaf senescence comprises numerous cooperative events, integrates environmental signals with age-dependent developmental cues, and coordinates the multifaceted deterioration and source-to-sink allocation of nutrients. In crops, leaf senescence has long been regarded as an essential developmental stage for productivity and quality, whereas functional characterization of candidate genes involved in the regulation of leaf senescence has, thus far, been limited in wheat.

**Results:**

In this study, we analyzed the expression profiles of 97 WRKY transcription factors (TFs) throughout the progression of leaf senescence in wheat and subsequently isolated a potential regulator of leaf senescence, TaWRKY42-B, for further functional investigation. By phenotypic and physiological analyses in *TaWRKY42-B*-overexpressing *Arabidopsis* plants and *TaWRKY42-B*-silenced wheat plants, we confirmed the positive role of TaWRKY42-B in the initiation of developmental and dark-induced leaf senescence. Furthermore, our results revealed that TaWRKY42-B promotes leaf senescence mainly by interacting with a JA biosynthesis gene, *AtLOX3*, and its ortholog, *TaLOX3*, which consequently contributes to the accumulation of JA content. In the present study, we also demonstrated that TaWRKY42-B was functionally conserved with AtWRKY53 in the initiation of age-dependent leaf senescence.

**Conclusion:**

Our results revealed a novel positive regulator of leaf senescence, TaWRKY42-B, which mediates JA-related leaf senescence via activation of JA biosynthesis and has the potential to be a target gene for molecular breeding in wheat.

## Background

Leaf senescence is a highly integrated developmental stage that coordinates multidimensional alterations at the physiological and molecular level [1]. During leaf senescence, intracellular organelles and macro-molecules are dismantled and degenerated, which predominantly contributes to the source-to-sink allocation of nutrients [2]. The redistribution of nutrients from senescent organs to vegetative tissues is essential for plant fitness and critical for productivity and quality in crops [3, 4]. Generally, the initiation of leaf senescence is age-dependent and triggered by developmental cues [5]. Additionally, leaf senescence is tightly controlled by epigenetic modification, circadian clock, autophagy, chlorophyll metabolism, and transcriptional regulation [6–17]. Meanwhile, when plants encounter abiotic and biotic stresses, the initiation of senescence will be accelerated [5, 18].

Phytohormones play both positive and negative roles in the onset of leaf senescence. Among the leaf senescence-related hormones, ethylene, abscisic acid (ABA), jasmonic acid (JA), salicylic acid (SA), brassinosteroids (BRs), and strigolactones (SLs) facilitate leaf senescence initiation, while cytokinins (CKs), gibberellic acid (GA), and auxins (AUXs) retard leaf senescence [19–28].

JA, a kind of lipid derived phytohormone, is vital for biotic stress defense, abiotic stress tolerance, and developmental regulation in plants [29, 30]. The functional role of JA in promoting leaf senescence was first identified by the severe degradation of chlorophyll in oat (*Avena sativa* L. cv “Victory”) leaves after application of methyl jasmonate (MeJA) in 1980 [31]. Meanwhile, the exogenous application of JA to wild-type *Arabidopsis* can elevate the transcripts of senescence-related genes, such as *SAG12* and *SEN4*, but not *coi1–1*. Moreover, expression of many JA biosynthesis genes and JA content are enhanced in senescent leaves [32]. A lipoxygenase for JA production, *AtLOX2*, is under the regulation of TCP4 in JA-induced leaf senescence, which is simultaneously repressed by *miRNA319* [33, 34]. Recently, studies on key components of the JA signaling pathway manifested that *Arabidopsis* MYC2, MYC3, and MYC4 promote leaf senescence by directly binding to the promoter of *SAG29*; this interaction is antagonistically regulated by III d group bHLHs [21]. However, the process of leaf senescence in *coi1–1* and some JA-related mutants is similar to the wild type (WT), suggesting that the dissection of the intricate mechanism underlying JA-induced leaf senescence is of immense importance [32, 35].

In addition to hormone responsive genes, numerous senescence-related genes, such as *senescence associated genes* (*SAGs*), have been proven to be crucial for the timing of leaf senescence [36]. Among which, senescence-related transcription factors, including NACs, WRKYs, bZIPs, MYCs, and MYBs, integrate various senescence signals into the nucleus and consequently execute transcriptional modulation [15, 16, 37]. WRKYs are named after the conserved WRKY domain that possesses a WRKYGQK core motif and a zinc-finger motif and is one of the biggest TF families in plants. Based on structural features and phylogenetic analysis, WRKYs are further divided into seven subgroups (I, IIa + IIb, IIc, IId + IIe, and III). To date, WRKYs are reported to participate in multiple stress-defense responses and developmental regulations. In addition, WRKYs involved in the modulation of leaf senescence have been widely acknowledged [38, 39]. Among those senescence-related WRKYs, AtWRKY53 has been intensively studied for its critical role in promoting leaf senescence initiation [40–42]. AtWRKY6 plays a role in GA- and light-related senescence processes [43–45]. AtWRKY57 coordinates JA and Aux signaling for fine tuning leaf senescence [46]. AtWRKY54 and AtWRKY70, two functionally redundant WRKYs, function as negative regulators in leaf senescence [47]. AtWRKY45 and AtWRKY75 trigger the initiation of leaf senescence via the GA and SA signaling pathway, respectively [22, 26]. Collectively, WRKYs are competent to integrate diverse signals for promoting and retarding leaf senescence onset in *Arabidopsis*. However, how WRKYs regulate leaf senescence in crops is still largely unknown.

Bread wheat (*Triticum aestivum* L.) is one of the most widely cultivated crops and serves as a staple food resource for more than 30% of the world’s population, providing approximately 20% of the calories consumed by humans [48]. The allohexaploid genome of bread wheat (AABBDD) arises from two polyploidization events. The first hybridization between diploid progenitors *Triticum urartu* (2n = 2x = 14, genome AA) and the section Sitopsis (donor of genome BB, presumably related to *Aegilops speltoides*, 2n = 2x = 14, genome SS) resulted in the formation of the allotetraploid ancestor *Triticum turgidum* (2n = 4x = 28; genome AABB). The second hybridization between *Triticum turgidum* and *Aegilops tauschii* (2n = 2x = 14, genome DD) finally contributed to the origination of bread wheat [49, 50]. Due to the allohexaploid nature of bread wheat, the sequencing of the genome and the characterization of functional genes have lagged behind those of other crops. Fortunately, databases established by next-generation sequencing technologies and whole-genome shotgun sequencing provide new approaches for wheat genome study [51–54]. However, candidate genes in the regulation of leaf senescence remain elusive and need to be explored to improve productivity and grain quality in wheat.

In this study, we aimed to screen the candidate genes of leaf senescence by an RNA-sequencing (RNA-seq) experiment in wheat. Consequently, we selected a WRKY type TF, TaWRKY42-B, for further functional research. Intriguingly, despite the expression levels of *TaWRKY42-B*, it was negatively correlated with the progression of leaf senescence, the silencing of *TaWRKY42-B* drastically delayed the leaf senescence process in wheat, and the overexpression of *TaWRKY42-B* in *Arabidopsis* caused premature leaf senescence, implying that TaWRKY42-B functioned as a positive regulator of leaf senescence. Subsequently, we demonstrated that TaWRKY42-B modulated leaf senescence was related to JA signaling through physiological and molecular analysis. We further manifested that TaWRKY42-B can promote JA biosynthesis by interacting with a JA biosynthesis gene *AtLOX3* and its ortholog (*TaLOX3*, *TraesCS4B02G295200*), altering the transcription of *AtLOX3* and *TaLOX3*. Moreover, the TaWRKY42-B-*TaLOX3* module underwent continuous suppression after leaf senescence onset, which possibly contributed to the organized progression of leaf senescence. Through complementary assay, we also showed that TaWRKY42-B had a partially conserved function with AtWRKY53 in the regulation of age-dependent leaf senescence. Briefly, our findings revealed the functional role of a new senescence-related TF, TaWRKY42-B, which is a positive and JA-related regulator of leaf senescence in wheat. Our data also suggest that TaWRKY42-B potentially serves as a target gene for molecular breeding in wheat.

## Results

### *TaWRKY42-B* is downregulated during leaf senescence

**Fig. 1:**
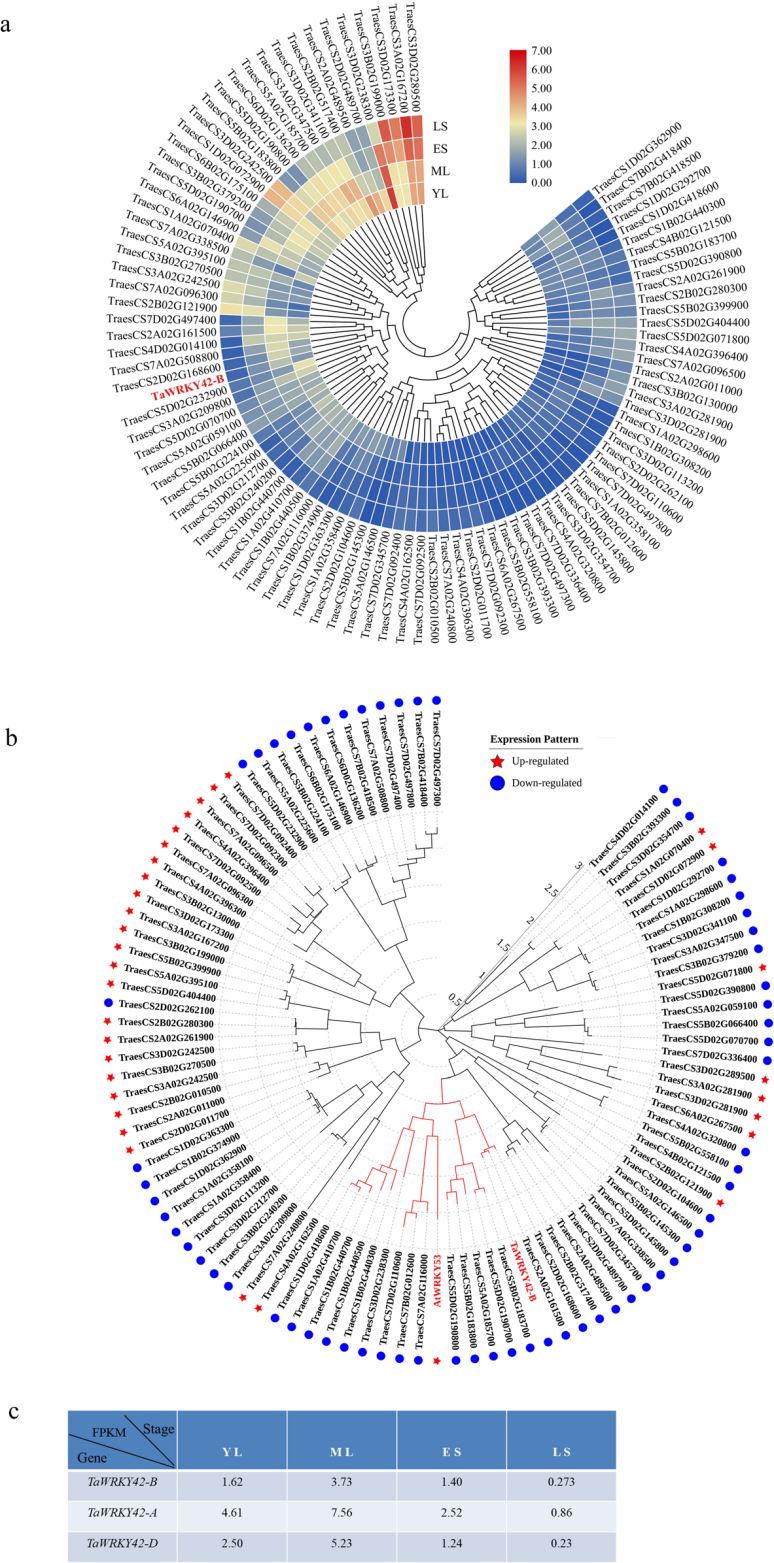
Expression profiling of *TaWRKY*s at different senescence stages. **a** Heatmap depiction of *TaWRKYs* among four developmental leaf stages. Blue indicates the expression level of *TaWRKYs* decreased relative to YL, whereas red indicates the transcription level of *TaWRKYs* relative to YL. (YL, young leaves; ML, mature leaves; ES, early senescent leaves, with < 25% leaf area yellowing; LS, late senescent leaves with > 50% leaf area yellowing). **b** Phylogenetic analysis of senescence-related TaWRKYs. **c** Transcription level (FPKM value) of *TaWRKY42-B*, *TaWRKY42-A* and *TaWRKY42-D* were monitored through the progression of leaf senescence. (Dots and asterisks indicate down and up-regulated genes)

**Fig. 2:**
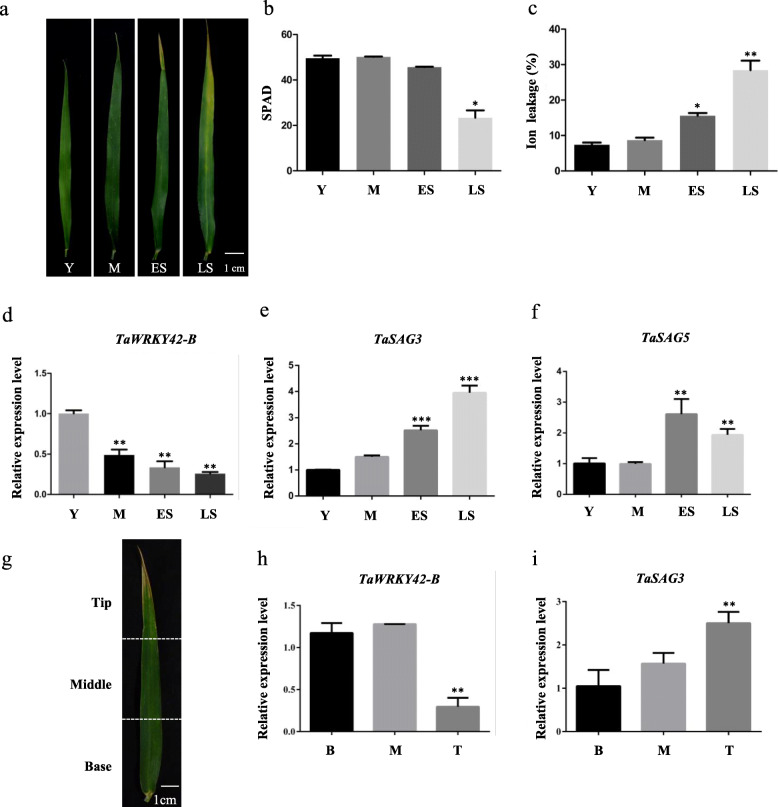
Spatiotemporal expression pattern of *TaWRKY42-B* in wheat. **a** Expression pattern of *TaWRKY42-B* was detected at four different developmental stages of wheat flag leaves (Y, M, ES, and LS). Chlorophyll content (**b**) and ion leakage rate (**c**) of leaves as shown in (**a**). **d**-**f** Transcription level of senescence-associated genes *TaWRKY42-B, TaSAG3* and *TaSAG5* in leaves as shown in (**a**). **g**-**i** Expression level of *TaWRKY42-B* and *TaSAG3* in tip, middle and base of a senescent wheat flag leaf. (Error bars indicate standard deviation (SD). Asterisks indicate significant differences. Student’s *t-*test, *P < 0.05, **P < 0.01, ***P < 0.001)

**Fig. 3:**
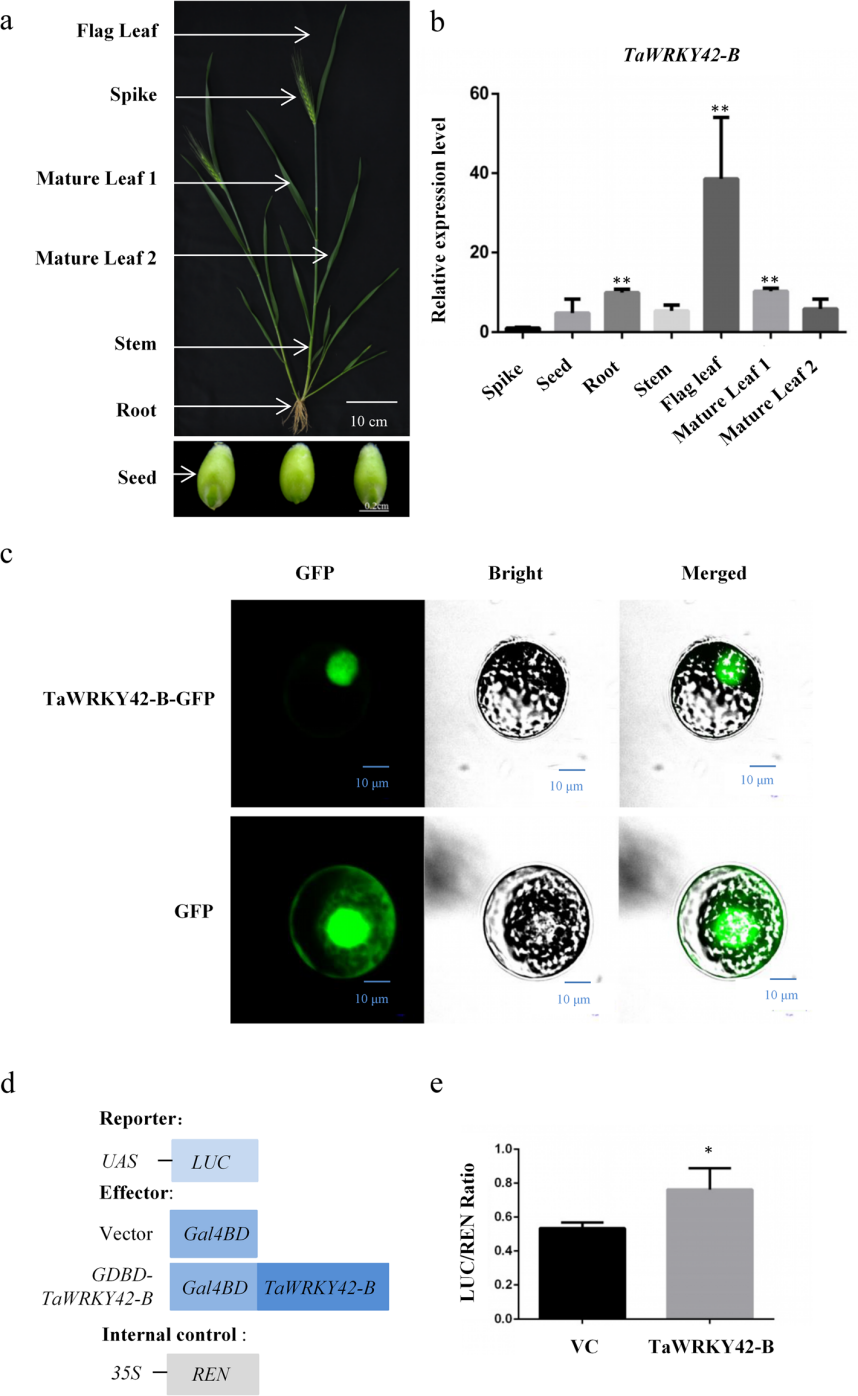
Tissue and subcellular expression pattern of *TaWRKY42-B* in wheat. **a**-**b** Tissue expression pattern of *TaWRKY42-B* was examined in different tissues (Flag leaf, Spike, 1st mature leaf from the top, Mature leaf 1; 2nd mature leaf from the top, Mature leaf 2; Stem, Root, and Seed) of 6-month-old wheat plants by qRT-PCR. **c** Subcellular localization of TaWRKY42-B-GFP fusion protein in the wheat protoplast. The control experiment employed a simple GFP protein. Bars = 10 μm. All experiments were repeated at least three times. **d** Diagrams of constructs in the transcriptional activity assay. **e** Measurement of relative LUC reporter activity after transient expression of vectors as shown in (**d**). (Error bars indicate SD. Asterisks indicate significant differences. Student’s *t*-test, *P < 0.05, **P < 0.01)

To identify candidate genes in the regulation of leaf senescence in *T. aestivum* (cv Chinese Spring), we conducted an RNA-seq experiment at four developmental stages of flag leaves (YL, young leaves; ML, mature leaves; ES, early senescent leaves; LS, late senescent leaves). For each sample, three biological replicates were employed. At senescent stages, we identified 32 up-regulated *WRKYs* and 65 down-regulated *WRKYs* compared with young leaf stage (|log2(fold change)| ≥1;*P* < 0.05) (Fig. 1a-b). Among the senescence-related *TaWRKYs* in this study, *TaWRKY42-B* (TraesCS2B02G187500), a member of the group III *WRKYs* (Additional file 1: Fig. S1a), exhibited the maximum expression level at ML stage and the decreasing expression through the progression of leaf senescence in flag leaves (Fig. 1c). To verify the expression pattern of *TaWRKY42-B*, we inspected the spatial-temporal transcriptional profiling of *TaWRKY42-B* in wheat. First, the negative correlation between transcription of *TaWRKY42-B* and age-dependent senescence process in wheat flag leaves was confirmed by quantitative real time-PCR (qRT-PCR) assay (Fig. 2d). Leaf senescence-associated parameters including chlorophyll content and ion leakage rate (Fig. 2b, c), as well as the transcription level of two *SAGs*, *TaSAG3* and *TaSAG5* (Fig. 2e, f), were examined for the propriety of harvesting flag leaves at distinctive senescence stages (Fig. 2a). Generally, senescence proceeded from the leaf tip and gradually progressed toward the leaf base [55] (Fig. 2g). Consistently, fewer transcripts of *TaWRKY42-B* were detected in the leaf tip than the middle and base (Fig. 2h), which was opposite to that of *TaSAG3* (Fig. 2i). Next, the transcription level of *TaWRKY42-B* was analyzed among different tissues, including spike, seed, root, stem, flag leaf, and mature leaf (Fig. 3a). *TaWRKY42-B* exhibited a ubiquitous expression pattern in wheat, whereas the most abundant transcripts of *TaWRKY42-B* were detected in flag leaves (Fig. 3b). The above data suggested that *TaWRKY42-B* is one of the *senescence down-regulated genes* (*SDGs*) and may predominantly function in leaves.

### TaWRKY42-B plays a positive role in age-dependent and dark-induced leaf senescence

**Fig. 4:**
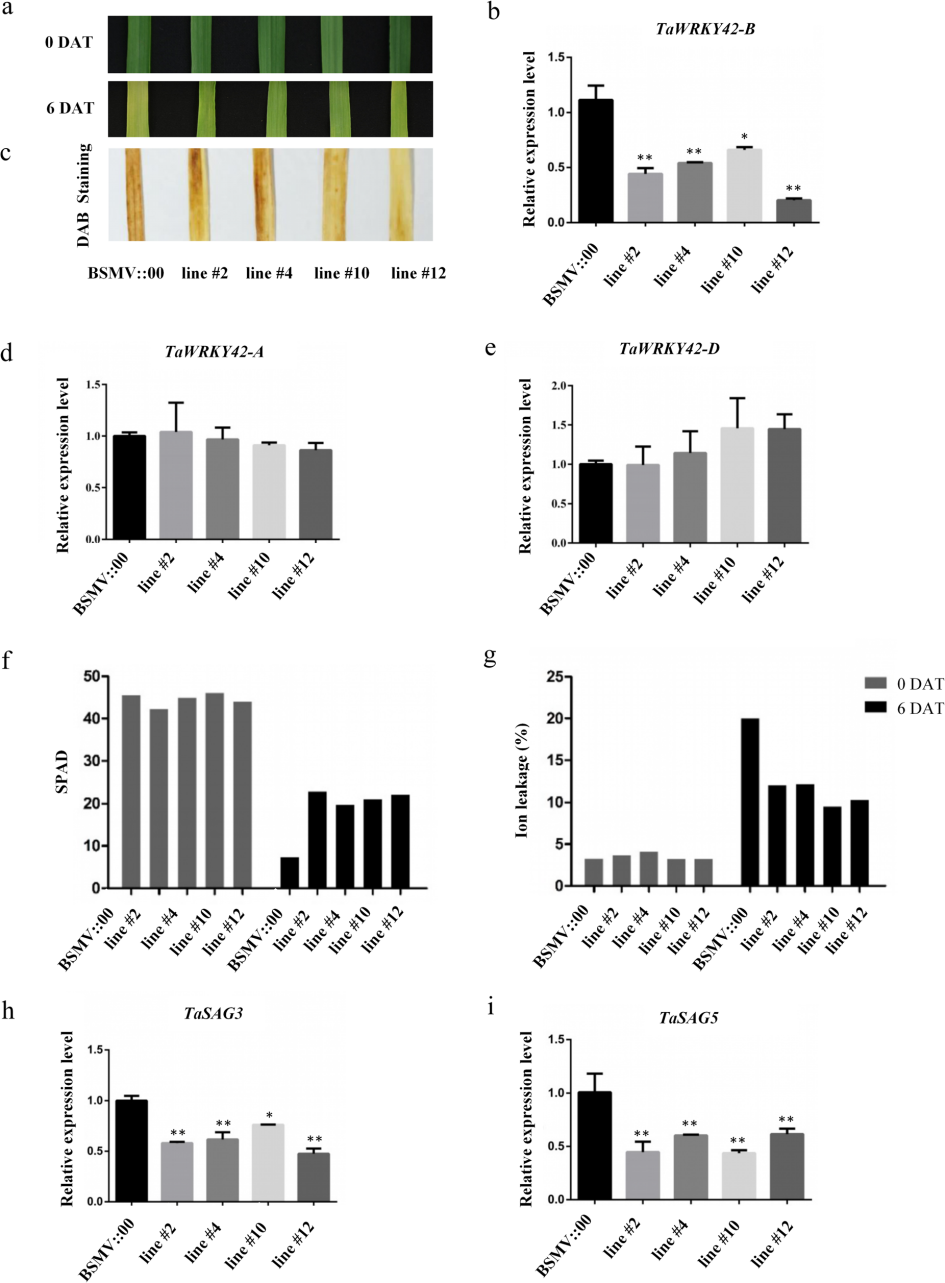
Silencing of *TaWRKY42-B* delays leaf senescence in wheat. **a** Phenotypic analysis of detached leaves of *TaWRKY42-B*-silenced plants and controls under darkness for 6 days. (DAT, Day after treatment). **b** Transcription level of *TaWRKY42-B* in leaves of *TaWRKY42-B*-silenced plants and controls. **c** DAB staining of detached leaves after dark treatment as shown in (**a**). **d**-**e** The expression level of *TaWRKY42-B* orthologs (*TaWRKY42-A* and *TaWRKY42-D*) in detached leaves of *TaWRKY42-B*-silenced plants and controls. Chlorophyll content (**f**) and ion leakage rate (**g**) of detached leaves as shown in (**a**). **h**-**i** Transcription level of senescence-associated genes *TaSAG3*, and *TaSAG5* of detached leaves as shown in (**a**)*.* (Error bars indicate SD. Asterisks indicate significant differences. Student’s *t*-test, *P < 0.05, **P < 0.01)

To further functionally characterize TaWRKY42-B, *TaWRKY42-B* was silenced by the *Barley stripe mosaic virus* (BSMV)-VIGS method in wheat with a 198 bp target sequence specifically selected from 679 bp downstream of the translation start codon of the *TaWRKY42-B* coding sequence (CDS) but not in *TaWRKY42-A* and *TaWRKY42-D* (Additional file 1: Fig. S1b, c and Additional file 2: Fig. S2a), and the empty vector (pCaBS-γbLIC) was used as a control. Seven-day-old wheat seedlings were infected with BSMV that harbored BSMV::TaWRKY42-B_198_ (pCaBS-α, pCaBS-β, and pCaBS-γbTaWRKY42-B_198_) (Additional file 2: Fig. S2b). After growth for another 25 days, the 8th leaf from the top of the vector control (VC) and *TaWRKY42-B*-silenced plants were detached and incubated in the dark for 6 days. *TaWRKY42-B-*silenced leaves exhibited an obviously delayed leaf senescence phenotype compared to VC transgenic leaves (Fig. 4a). Consistently, chlorophyll degradation, ion leakage rate, and expression of *TaSAG3* and *TaSAG5* in *TaWRKY42-B-*silenced leaves were remarkably decreased compared with those of VC plants (Fig. 4f-i). Accumulation of hydrogen peroxide (H_2_O_2_) in senescent leaves has long been known for the subsequent oxidative damage. Less dark brown spots existed on *TaWRKY42-B-*silenced leaves after 3,3-diaminobenzidine (DAB) staining, which demonstrated that the senescence process was delayed by silencing of *TaWRKY42-B* (Fig. 4c). To estimate whether the delayed leaf senescence resulted from the depressed expression level of *TaWRKY42-B*, the transcription level of *TaWRKY42-A*, *TaWRKY42-B*, and *TaWRKY42-D* was examined in those infected wheat plants. Notably, the stay-green phenotype was clearly observed after dark treatment in detached leaves with diminished transcription of *TaWRKY42-B* (Fig. 4b) and comparable levels of *TaWRKY42-A* and *TaWRKY42-D* to that of the control leaves (Fig. 4d, e). This result suggested that TaWRKY42-B was a positive regulator of leaf senescence onset in wheat.

**Fig. 5:**
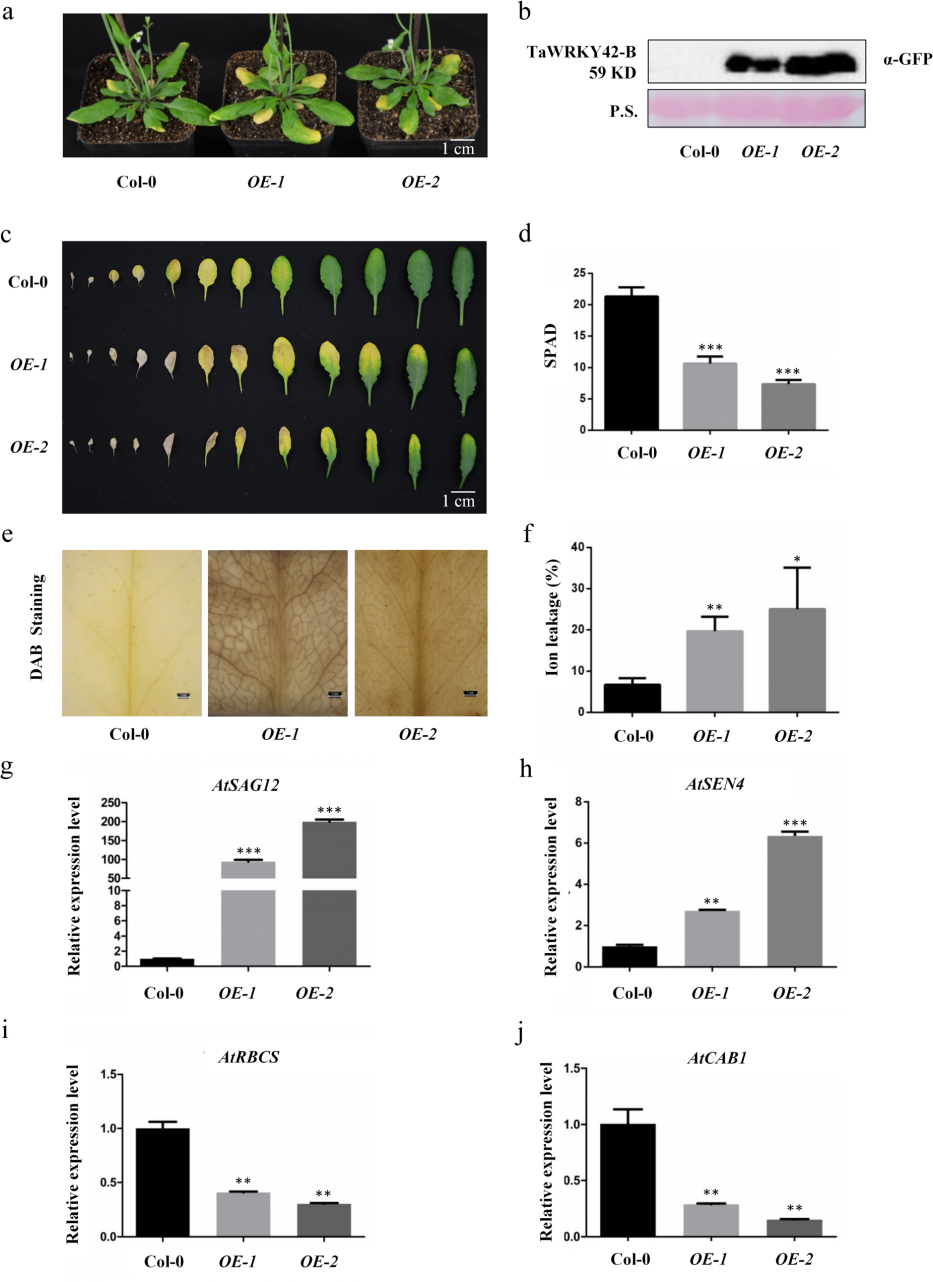
Overexpression of *TaWRKY42-B* accelerates leaf senescence in *Arabidopsis*. **a** Phenotypic analysis of 6-week-old Col-0 and *TaWRKY42-B*-overexpressing plants. **b** Protein level of TaWRKY42-B in Col-0 and *TaWRKY42*-*B*-overexpressing plants. Ponceau S (P.S.) staining indicates equal loading. **c** Detached rosette leaves of 6-week-old Col-0 and transgenic plants were laid out. Chlorophyll content (**d**) and ion leakage rate (**f**) of the 6th and 7th detached leaves in (**c**). **e** DAB staining of 4-week-old Col-0 and *TaWRKY42-B-*overexpressing plants. **g**-**j** Detection of expression level of senescence-associated genes *AtSAG12*, *AtSEN4*, *AtRBCS*, and *AtCAB1* in 4-week-old Col-0 and *TaWRKY42-B-*overexpressing plants by qRT-PCR. (Error bars indicate SD. Asterisks indicate significant differences. Student’s *t*-test, *P < 0.05, **P < 0.01, ***P < 0.001)

To further assess the functional role of *TaWRKY42-B* in leaf senescence, *TaWRKY42-B*-overexpressing (OE) *Arabidopsis* plants were generated. A full-length 879 bp CDS of *TaWRKY42-B* was cloned into pCAMBIA1300 and fused with green fluorescent protein (GFP) tag, which was driven by the *Cauliflower mosaic virus* (CaMV) 35S promoter. Two independent homozygous transgenic lines (*OE-1* and *OE-2*) were selected for further phenotypic and physiological analysis. The elevated expression level of TaWRKY42-B was confirmed by a western blot in *OE-1* and *OE-2* (Fig. 5b). Next, we observed that 5-week-old *OE-1* and *OE-2* plants showed a precocious leaf senescence phenotype compared with Col-0 (Fig. 5a, c). The chlorophyll content of *TaWRKY42-B*-*OE* lines decreased in Col-0 (Fig. 5d) and ion leakage rates were accelerated by overexpression of *TaWRKY42-B* (Fig. 5f). More H_2_O_2_ was detected by DAB staining in *TaWRKY42-B*-*OE* lines (Fig. 5e). Consistent with the observed phenotypic alterations, two *SAGs*, *AtSAG12* and *AtSEN4*, were upregulated in *OE-1* and *OE-2* (Fig. 5g, h), while two *SDGs*, *AtRBCS* and *AtCAB1*, were suppressed in *TaWRKY42-B*-*OE* (Fig. 5i, j). To investigate whether *TaWRKY42-B* was involved in dark-induced leaf senescence in *Arabidopsis*, we detached the 8th rosette leaves from *OE-1*, *OE-2*, and Col-0, which were apparently undistinguishable and subsequently, incubated under darkness for another 6 days (Additional file 5: Fig. S5a). Senescence-related phenotypic alterations, chlorophyll degradation and ion leakage rate were more dramatically changed in *OE-1* and *OE-2* after dark treatment (Additional file 5: Fig. S5b, c). The above data validated that TaWRKY42-B promoted leaf senescence under both optimal growth conditions and darkness.

### TaWRKY42-B localizes in the nucleus and possesses transcriptional activity

Generally, WRKYs are implicated in transcriptional regulation of diverse biological processes. To illustrate whether TaWRKY42-B plays a role in transcriptional modulation, we produced a *35S:TaWRKY42-B-GFP* construct, which was then transformed and transiently expressed into wheat protoplasts. GFP fluorescence signals of TaWRKY42-B-GFP fusion predominantly concentrated in the nucleus, while signals of GFP alone were visualized in the plasma membrane, cytoplasm, and nucleus (Fig. 3c). This specific subcellular localization of TaWRKY42-B implied that TaWRKY42-B potentially participated in transcriptional regulation in wheat. TaWRKY-42-B showed strong transcriptional activity in yeast (Additional file 3: Fig. S3). Consequently, the transcriptional activity of TaWRKY42-B in wheat protoplasts was detected by a dual-luciferase reporter system. TaWRKY42-B was fused to a Gal4 DNA-binding domain (GDBD) and co-transfected with the *firefly luciferase* (*LUC*) gene, which was under the control of an upstream activating sequence (UAS) and the *Renilla luciferase* (*REN*) gene, driven by a CaMV 35S promoter fusion was used as an internal control (Fig. 3d). After the addition of enzymatic substrates, GDBD-TaWRKY42-B, but not GDBD only, was able to facilitate the enzymatic reaction of LUC (Fig. 3e). These results indicated that TaWRKY42-B was a nucleus-localized TF.

### JA is essential for TaWRKY42-B to trigger leaf senescence

**Fig. 6:**
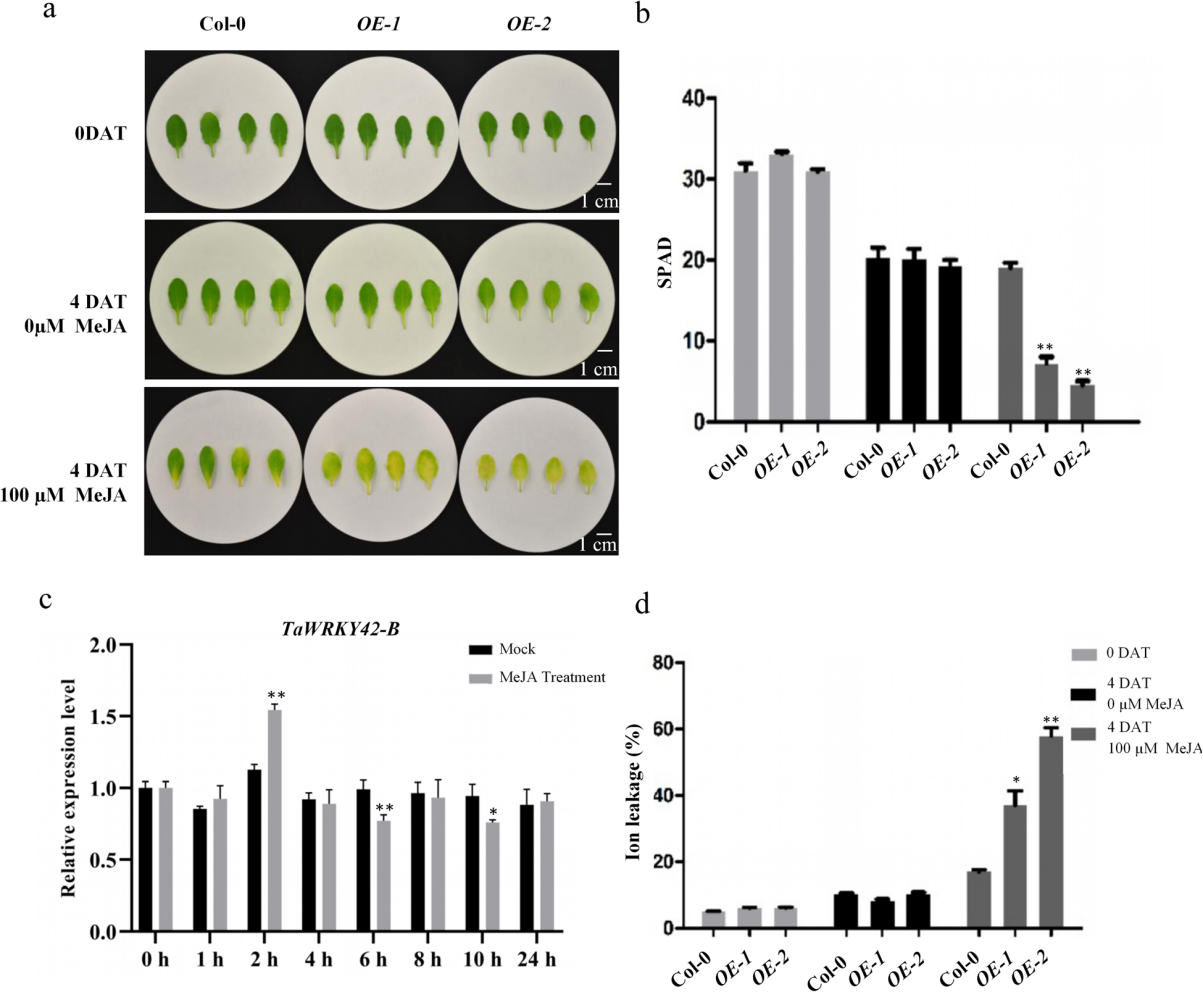
TaWRKY42-B positively regulates JA-induced leaf senescence in *Arabidopsis*. (**a**) Senescence-related phenotypes of 7th and 8th leaves of 4-week-old Col-0 and *TaWRKY42-B*-overexpressing (*TaWRKY42-B-*OE) plants before and after treated with mock or 100 μM MeJA. Chlorophyll content (**b**) and ion leakage rate (**d**) of detached leaves before and after treatment as shown in (**a**). (**c**) Transcription of *TaWRKY42-B* was examined after MeJA treatment in wheat. Seven-day-old wheat seedlings were treated with 200 μM MeJA for 1, 2, 4, 6, 8, 10 and 24 h. (Error bars indicate SD. Asterisks indicate significant differences. Student’s *t*-test, *P < 0.05, **P < 0.01)

**Fig. 7:**
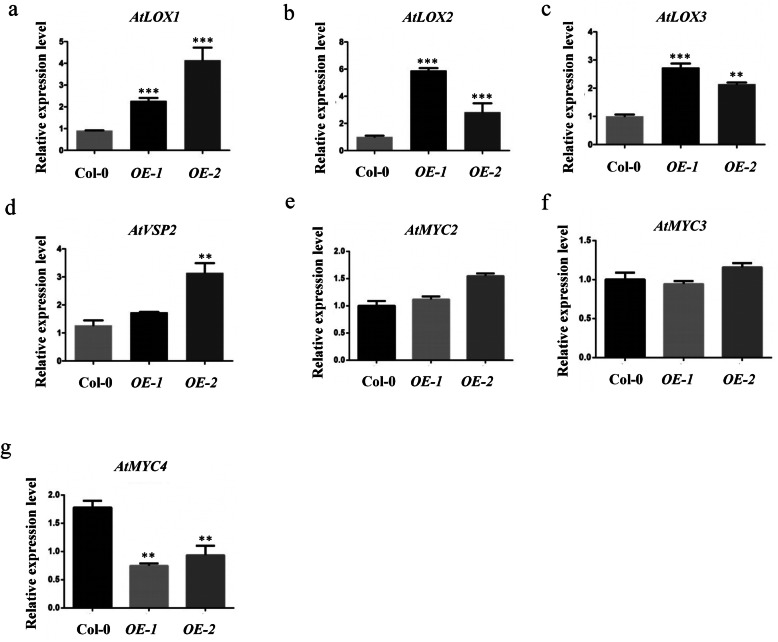
TaWRKY42-B affects expression of JA-related genes in *Arabidopsis*. **a**-**g** The transcription level of several JA-related genes, including *AtLOX1*, *AtLOX2*, *AtLOX3*, *AtVSP2*, *AtMYC2*, *AtMYC3*, and *AtMYC4*, in the 7th and 8th rosette leaves of 4-week-old *TaWRKY42-B-OE* and Col-0 plants detected by qRT-PCR. (Error bars indicate SD. Asterisks indicate significant differences. Student’s *t*-test, *P < 0.05, **P < 0.01, ***P < 0.001)

To uncover the mechanism underlying TaWRKY42-B-induced leaf senescence, we searched *cis*-acting elements that were distributed in the *TaWRKY42-B* promoter region for clues. Noticeably, three CGTCA-motifs related to JA responsiveness lay in the *TaWRKY42-B* promoter region (− 980, − 1865, and − 2009 bp) (Additional file 4: Fig. S4). In addition, after the application of 200 μM MeJA, transcription levels of *TaWRKY42-B* were at a maximum after 2 h and then decreased to a relatively low level from 4 to 24 h (Fig. 6c). For decades, JA has been well acknowledged as a leaf senescence-related phytohormone. Therefore, we speculated that TaWRKY42-B modulated leaf senescence in a JA-related manner. Rosette leaves were harvested from Col-0, *OE-1*, and *OE-2* and treated with 100 μM MeJA for 4 days. JA-induced leaf senescence appeared in both Col-0 and *TaWRKY42-B*-*OE* plants, but lower chlorophyll content and more severe ion leakage were found only in *OE-1* and *OE-2* (Fig. 6a, b, and d). To finely dissect the details of the interaction between TaWRKY42-B and the JA signaling pathway, the expression level of a series of JA-related genes were inspected. JA biosynthesis genes, including *AtLOX1*, *AtLOX2*, *AtLOX3*, and a JA-responsive gene, *AtVSP2*, were elevated in *OE-1* and *OE-2* when compared to Col-0 (Fig. 7a-d). However, signaling component genes, such as *AtMYC2* and *AtMYC3,* were nearly unaffected by overexpression of *TaWRKY42-B* (Fig. 7e-f). Meanwhile, the expression of *AtMYC4* was slightly decreased in *TaWRKY42-B-OE* plants (Fig. 7g). These results suggested that TaWRKY42-B promoted leaf senescence mainly by altering the expression of JA biosynthesis genes.

### TaWRKY42-B directly binds to the promoter region of *LOXs*

**Fig. 8:**
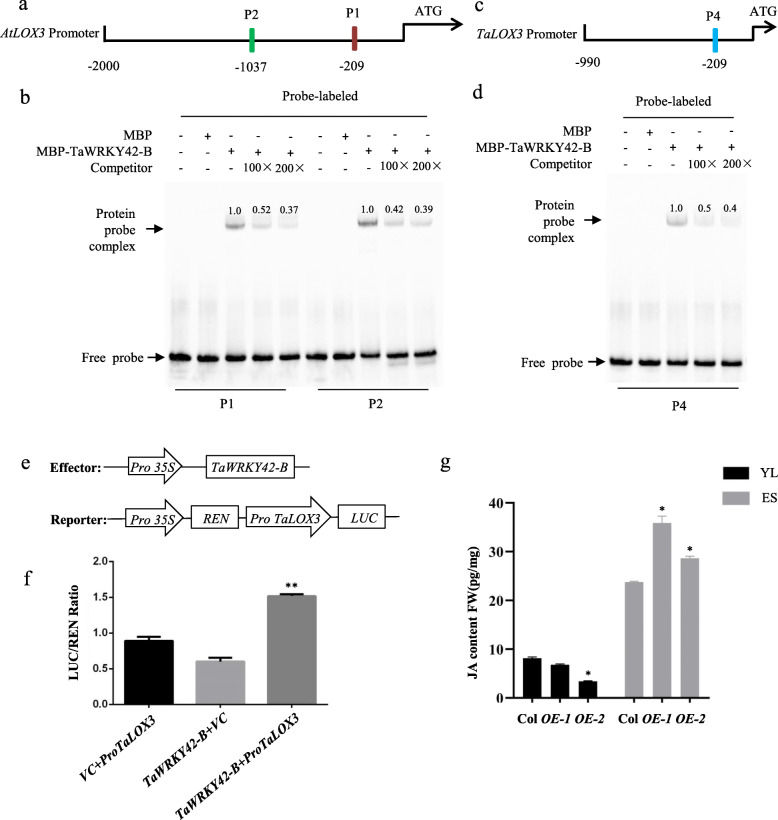
TaWRKY42-B directly binds to the promotor of *AtLOX3* and *TaLOX3*, affecting transcription of *TaLOX3*. **a** Diagram of probes against the *AtLOX3* promoter region for EMSA. **b** Interaction between TaWRKY42-B and the promoter region of *AtLOX3* analyzed by EMSA. **c** Diagram of probes against the *TaLOX3* promoter region for EMSA. **d** Interaction between TaWRKY42-B and the promoter region of *TaLOX3* analyzed by EMSA. TaWRKY42-B-MBP fusion protein mixed with labeled probes, with 100× or 200× unlabeled probes serving as competitors. The presence (+) or absence (−) of specific probes. Numbers above the top of the bands indicate the relative binding strength of the TaWRKY42-B-MBP fusion protein and labeled probes after normalization to the control group. **e** Diagrams of constructs in the transient expression assay. **f** TaWRKY42-B activated the expression of *TaLOX3* in dual-luciferase reporter system in wheat protoplasts. *Renilla* luciferase was used for normalization. *35S:GFP* act as a negative control. **g** Measurement of JA content in the 7th and 8th rosette leaves of three- and five-week-old Col-0 and *TaWRKY42-B-OE* lines, respectively. (Error bars indicate SD. Asterisks indicate significant differences in SPAD and ion leakage between the negative control and TaWRKY42-B group. Student’s *t*-test, *P < 0.05, **P < 0.01)

WRKYs have long been known to regulate the transcription of diverse target genes, which is generally accomplished by the interaction between WRKYs and W-box contained promoter regions. We identified one and two W-box motifs in *AtLOX1* and *AtLOX3* promoters, respectively (Additional file 6: Fig. S6a and Fig. 8a). Thus, we investigated whether TaWRKY42-B binds to the W-boxes in *AtLOX1* and *AtLOX3* promoters by an electrophoretic mobility shift assay [56]. The full-length CDS of TaWRKY42-B was subcloned into the frame of pMAL-c2x and fused to the Maltose Binding Protein (MBP). TaWRKY42-B-MBP fusion proteins were expressed and purified from *E. coli* strain *Rosetta*. Probes against the W-box containing fragments were incubated with TaWRKY42-B-MBP or MBP alone. Probe 1 (P1) and probe 2 (P2) contained the W-boxes of the *AtLOX3* promoter, specifically hybridized with TaWRKY42-B-MBP and were competed against unlabeled probes (Fig. 8b). Probe 3 (P3), which was used for testing whether TaWRKY42-MBP targeted *AtLOX1*, did not show competitive binding with TaWRKY42-B-MBP (Additional file 6: Fig. S6b). These data suggested that *AtLOX3* was a potential target gene of TaWRKY42-B in *Arabidopsis*.

**Fig. 9:**
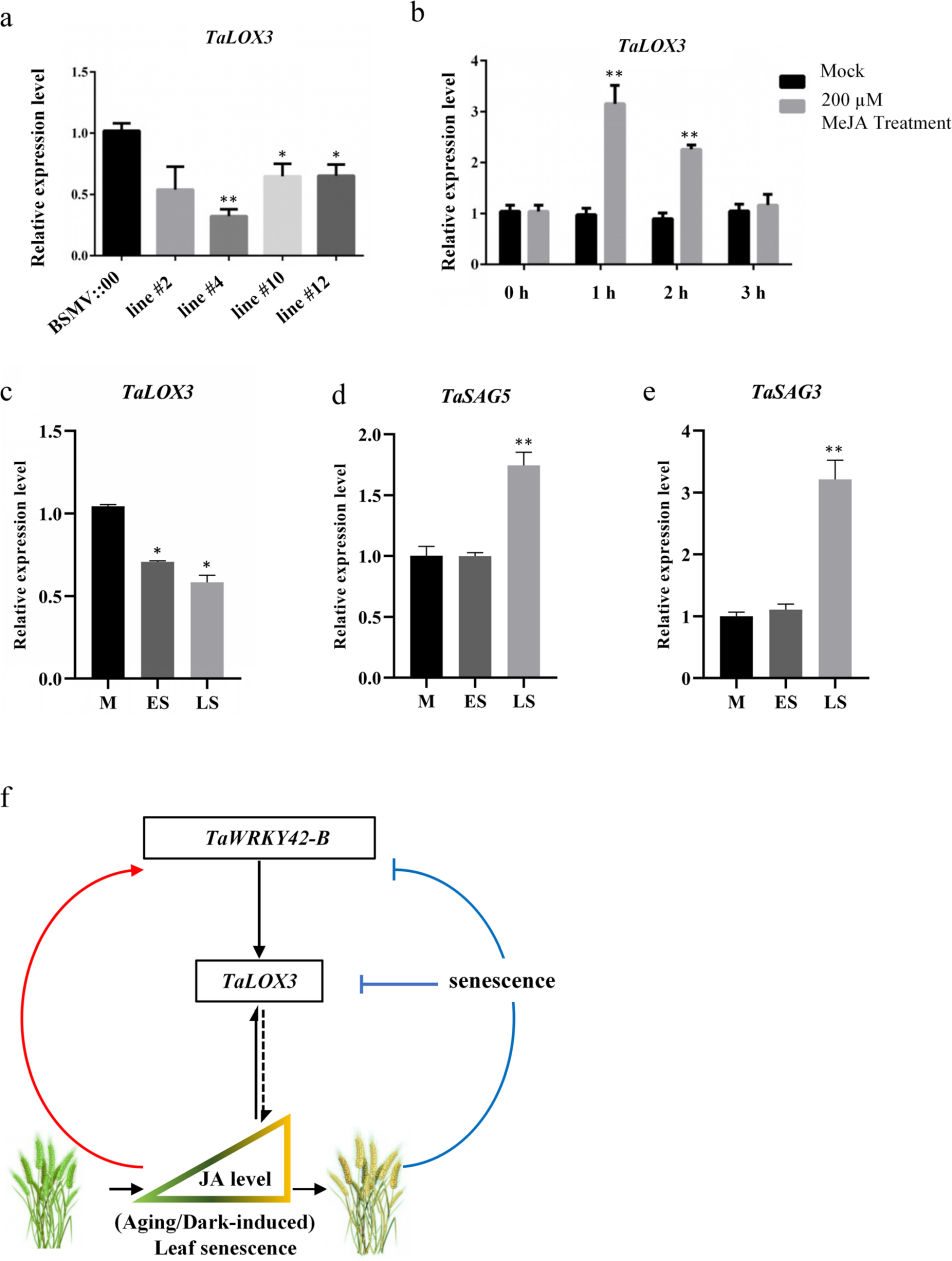
*TaLOX3* expression is altered by TaWRKY42-B, MeJA treatment, and the senescence process. **a** Expression level of *TaLOX3* in *TaWRKY42-B*-silencing plants. **b** Transcription of *TaLOX3* was detected after MeJA treatment. Seven-day-old wheat seedlings were treated with 200 μM MeJA for 1, 2 and 3 h. **c**-**e** Expression pattern of senescence-associated genes *TaSAG3*, *TaSAG5*, and *TaLOX3* at different developmental stages of wheat flag leaves (M, ES, and LS). **f** Proposed model of the TaWRKY42-B-mediated regulatory network of leaf senescence. TaWRKY42-B functions as a promoter of leaf senescence through activating a putative JA biosynthesis gene, *TaLOX3*, which is also induced by JA signaling. When plants are at the juvenile stage, high levels of *TaWRKY42-B* are likely responsible for JA-related developmental regulation and biosynthesis. While wheat plants advance into the senescent stage, leaf senescence suppresses the TaWRKY42-B-*TaLOX3* module for normal progression of leaf senescence. The feedback control of *TaWRKY42-B* by JA signaling, and leaf senescence has the potential to be a vital step of timing of leaf senescence. (Error bars indicate SD. Asterisks indicate significant differences. Student’s *t*-test, *P < 0.05, **P < 0.01)

Despite that TaWRKY42-B interacted with *AtLOX3*, little was known about the potential of TaWRKY42-B binding to the orthologs of *AtLOX3* in wheat. We used the *AtLOX3* genomic sequence as a query to search against the wheat genome database (http://plants.ensembl.org/Triticum_aestivum). A unique gene (*TraesCS4B02G295200*), sharing the highest similarity with *AtLOX3*, was termed *TaLOX3* and selected for testing. We searched the *TaLOX3* promoter region and found only a single W-box element existing within a 209 bp fragment upstream of the start codon (Fig. 8c). Subsequently, we tested the interaction between TaWRKY42-B and *TaLOX3* by EMSA. Consistent with the interaction between TaWRKY42-B and *AtLOX3*, probe 4 (P4) against the *TaLOX3* promoter strongly bound to TaWRKY42-B in vitro (Fig. 8d). To explore the relationship between TaWRKY42-B and *TaLOX3* in vivo, the enzymatic activity of LUC, which was under the control of the *TaLOX3* promoter and co-transformed with *35S:TaWRKY42-B* into wheat protoplasts, was measured (Fig. 8e). The drastic elevation of LUC activity was only observed in protoplasts harboring both *35S:TaWRKY42-B* and *P*_*TaLOX3*_*:LUC* but not in the control groups (Fig. 8f). Additionally, we analyzed the expression level of *TaLOX3* in TaWRKY42-B-silenced plants, and *TaLOX3* was remarkably depressed by silencing *TaWRKY42-B* (Fig. 9a).

Noticeably, the functional role of *TaLOX3* was still uncharacterized. Thus, to preliminarily illuminate whether TaLOX3 played a role in JA-induced leaf senescence, we checked the transcripts of *TaLOX3* in 7-day-old wheat seedlings, which were treated by 200 μM MeJA for 3 h. By the qRT-PCR assay, we illustrated that MeJA was able to enhance the expression of *TaLOX3* (Fig. 9b). In addition, decreasing expression of *TaLOX3* was detected from the mature leaf stage to the late senescent leaf stage, which is basically in line with the expression pattern of TaWRKY42-B and inverse to the expression of *TaSAG3* and *TaSAG5* (Fig. 9c-e). Briefly, the above data implied that TaLOX3 is possibly involved in JA-induced leaf senescence in wheat; however, more evidence is still necessary for a comprehensive understanding of the functional role of TaWRKY42-B-*TaLOX3* module in wheat leaf senescence.

### TaWRKY42-B promotes JA biosynthesis

To further confirm that TaWRKY42-B facilitated leaf senescence via JA biosynthesis, the JA level was measured in young and senescent leaves of *TaWRKY42-B*-*OE* and wild-type *Arabidopsis* by LC-MS/MS, respectively. Consistent with previous studies, the JA level was increased throughout the progression of leaf senescence. Notably, the JA level in *TaWRKY42-B*-OE plants was lower than that in the wild type at the juvenile stage but higher than that of wild-type plants at the senescent stage (Fig. 8g). Based on this data, we speculated that appropriate transcriptional abundance of *TaWRKY42-B* partially contributed to the homeostasis of JA biosynthesis at different developmental stages, hence the expression level of *TaWRKY42-B* should be tightly controlled.

### TaWRKY42-B is conserved with AtWRKY53 in age-dependent leaf senescence

**Fig. 10:**
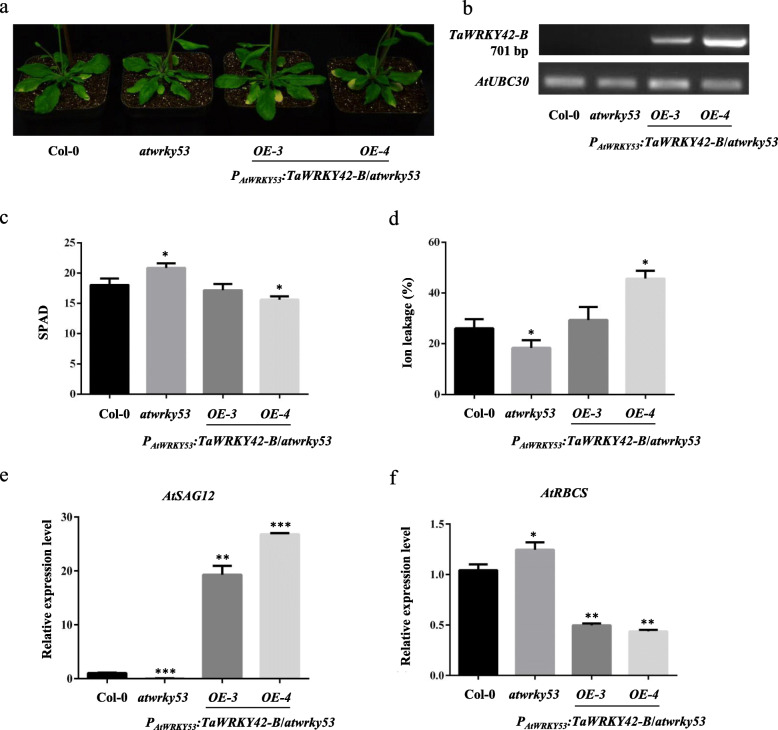
*TaWRKY42-B* recovers the delayed leaf senescence phenotype in *wrky53*. **a** Senescence-related phenotype of 6-week-old Col-0, *atwrky53*, and *wrky53/WRK42-B-OE* lines. **b** Reverse transcription PCR (RT-PCR) analysis of *TaWRKY42-B* in *P*_*AtWRKY53*_*:TaWRKY42-B*/*atwrky53*, *atwrky53*, and Col-0 plants. Chlorophyll content (**c**) and ion leakage rate (**d**) of different background plants as shown in (**a**). **e**-**f** Transcription level of *AtSAG12* and *AtRBCS* of different background plants as shown in (**a**). (Error bars indicate SD. Asterisks indicate significant differences. Student’s *t*-test, *P < 0.05, **P < 0.01, ***P < 0.001)

AtWRKY53, as a key regulator of the leaf senescence process, integrated various environmental and internal factors for ultimately initiating leaf senescence. In our study, phylogenetic analysis and sequence alignment indicated that TaWRKY42-B was a member of the group III WRKYs, which also comprises AtWRKY53. To validate whether TaWRKY42-B was functionally conserved with AtWRKY53, a complementation experiment was performed, for which CDS of *TaWRKY42-B* driven by the 1.5 kb *AtWRKY53* promoter was introduced into the *atwrky53* mutant (Fig. 10b). The delayed leaf senescence phenotype in *atwrky53* was rescued by the expression of *TaWRKY42-B* (Fig. 10a). Consistently, chlorophyll content, ion leakage rate, and expression level of *AtSAG12* and *AtRBCS* in *P*_*AtWRKY53*_*:TaWRKY42-B*/*atwrky53* lines was restored (Fig. 10c-f). These data manifested that the functional role of TaWRKY42-B was conserved with AtWRKY53 in the regulation of age-dependent leaf senescence.

## Discussion

Senescence is not only the final developmental stage for plants but is also essential for their productivity and fitness, which has special significance in crops [4]. As leaf senescence proceeds, nitrogen relocates from senescent leaves to grains, which accounts for around 95% of the total nitrogen in crop grains [57–59]. Improper timing of leaf senescence caused by biotic and abiotic stresses and abnormal developmental events is detrimental to diverse biological processes. Thus, leaf senescence must be a highly ordered program and tightly controlled by a variety of senescence-related genes. The delicate modulation of leaf senescence governed by TFs has been studied for decades and is regarded as a key step for integrating various senescence-related signals.

WRKYs are one of the largest TF families in plants, playing critical roles in developmental regulation and stress defense responses [39, 60]. Recently, some WRKYs involved biological processes have been studied in wheat. TaWRKY51 affected ethylene production and subsequently promoted lateral root formation [61]. TaWRKY2 was induced by drought, salt, heat, and ABA, and overexpression of *TaWRKY2* led to enhanced tolerance of transgenic wheat to drought stress and increased yield [62, 63]. TaWRKY19 positively regulated salt, drought, and freezing stress responses in *Arabidopsis*, which was partially due to the elevated expression of *DREB2A*, *RD29A*, *RD29B*, and *Cor6.6* by TaWRKY19 [63]. Silencing of *TaWRKY70* downregulated the transcription of metabolite biosynthetic genes and thus, decreased the resistance to *Fusarium graminearum* in wheat [64]. Noticeably, WRKYs are involved in the regulation of leaf senescence in different plant species, but senescence-related WRKYs are largely unknown in wheat.

In the present study, we screened the leaf senescence-related TaWRKYs by an RNA-seq experiment. Intriguingly, the transcription level of *TaWRKY42-B* deceased throughout the progression of leaf senescence, however, *TaWRKY42-B* played a positive role in the initiation of leaf senescence. This special characteristic of TaWRKY42-B led us to further investigate the functional role of TaWRKY42-B in the regulation of leaf senescence. Similarly, two *Arabidopsis* regulators of leaf senescence onset, *AtWRKY54* and *AtWRKY70*, are basically accumulated during leaf senescence but they are partially redundant in delaying leaf senescence [47]. Moreover, *AtJUB1* delayed leaf senescence initiation and exhibited an age-dependent elevation at the transcriptional level [65]. Previous research partly illustrated that the timing of leaf senescence was orchestrated by a variety of regulators. Thus, the dissection of the sophisticated underlying mechanisms of both senescence-upregulated and -downregulated TFs in the modulation of leaf senescence are of immense importance.

Subsequently, we confirmed the positive role of TaWRKY42-B in the onset of leaf senescence by overexpression of *TaWRKY42-B* in Arabidopsis. Meanwhile, BSMV-VIGS was a powerful and convenient tool for functional characterization of genes, especially helpful for some crops that are difficult for stable transformation. Using BSMV-VIGS, we found that the onset of leaf senescence was remarkably retarded in wheat plants with a declined expression level of *TaWRKY42-B* but not *TaWRKY42-A* and *TaWRKY42-D*. Notably, following the depression of the mRNA level of T*aWRKY42-B* upon BSMV-VIGS, transcripts of *TaWRKY42-D* and *TaWRKY42-A* presented slight alterations at times, which was possibly led by a compensatory effect and nonspecific silencing. To clarify the connection between *TaWRKY42-B* and leaf senescence onset in this study, we performed senescence-related phenotypic analyses after detecting the expression the levels of *TaWRKY42-B*, *TaWRKY42-A*, and *TaWRKY42-D* in those BSMV-VIGS wheat plants. Only the wheat plants with diminished transcripts of *TaWRKY42-B* and comparable levels of *TaWRKY42-A* and *TaWRKY42-D* compared to those of control plants were selected for further analyses. Thus, we concluded that the delayed leaf senescence phenotype in this study resulted from *TaWRKY42-B* silencing in wheat. Briefly, the potential of TaWRKY42-B in promoting leaf senescence in monocotyledons and dicotyledons suggested that TaWRKY42-B regulated leaf senescence through a conserved mechanism in plants.

By sequence alignment and phylogenetic analysis, we identified TaWRKY42-B as a member of group III WRKYs, which also comprises some senescence-related WRKYs, such as AtWRKY53. Based on reported studies, senescence-related TFs modulating the initiation of leaf senescence predominately relied on its transcriptional activity. To assess whether TaWRKY42-B impacted the timing of leaf senescence via transcriptional regulation, we examined the transcriptional activity of TaWRKY42-B in wheat protoplasts and yeast cells, by which TaWRKY42-B exhibited strong transcriptional activity in both analyses. Consistently, nucleus localization of TaWRKY42-B in wheat mesophyll cell protoplasts also endowed TaWRKY42-B with the potential to regulate the transcription of senescence-related genes.

Furthermore, we demonstrated that TaWRKY42-B affected the leaf senescence process through JA signaling. Leaf senescence was accelerated in *TaWRKY42-B*-overexpressing plants upon MeJA treatment. Additionally, we confirmed that TaWRKY42-B promoted the accumulation of JA in vivo. Noticeably, ectopic expression of *TaWRKY42-B* resulted in a lower and a higher JA level at the juvenile and senescent stage, respectively. This result suggested the pleiotropic roles of TaWRKY42-B at different stages. Hence, *TaWRKY42-B* should be tightly restricted to an appropriate level for normal growth. Consistently, we validated that TaWRKY42-B bound to the promoter regions of *AtLOX3* and *TaLOX3* by EMSA, which suggested that *TaLOX3* and *AtLOX3* are potential target genes of TaWRKY42-B. Further, by using dual-luciferase reporter system in wheat protoplasts, we demonstrated the activation of *TaLOX3* promoter by TaWRKY42-B. Moreover, *TaLOX3* showed a decreasing expression level from the mature leaf stage to the late senescent leaf stage, which coincides with the expression pattern of *TaWRKY42-B*. Collectively, we speculated that the positive correlation between transcription of *AtLOX3*, *TaLOX3*, and TaWRKY42-B was a consequence of the interaction of TaWRKY42-B with *AtLOX3* and *TaLOX3*. This modulation was likely promoted by JA accumulation and consequently, triggered the initiation of leaf senescence, although some convincing evidence, such as ChIP-PCR results, will be needed to further validate the physical interaction between TaWRKY42-B and *LOXs* in future.

JA has long been regarded as a senescence-related phytohormone [31]. Although the content of JA and JA biosynthesis genes are elevated in senescent leaves, the senescence process is identical in wild type and some JA-related mutants, which implies that the functional roles of JA signaling in leaf senescence still require consideration [32].

In wheat, a few JA-related genes have been characterized and functionally studied. TaJAZ1 directly interacted with TaABI5, which consequently led to the inhibition of seed germination [66]. TaJAZ1 also positively regulated the resistance to powdery mildew [67]. *TaAOC1*, a JA biosynthesis gene, was responsible for JA accumulation and tolerance to salinity [68]. *TaOPR2* functioned in JA biosynthesis and could rescue the male sterility phenotype of *opr3* in *Arabidopsis* [69]. However, identification of the regulators involved in JA-mediated leaf senescence in wheat is lagging behind many crops. The dramatically delayed leaf senescence phenotype of *TaWRKY42-B*-silenced wheat plants and precocious leaf senescence in *TaWRKY42-B*-overexpressing *Arabidopsis* manifested that TaWRKY42-B had a positive role in the onset of leaf senescence. Our data also suggested that TaWRKY42-B promoted leaf senescence via the JA pathway. Intriguingly, TaWRKY42-B was a promoter of the leaf senescence onset but with a decreasing expression pattern as leaf senescence proceeded. To explain the functional role of TaWRKY42-B, we proposed a hypothesis that is mainly based on the effect of TaWRKY42-B on JA biosynthesis in leaf senescence. We speculated that *TaWRKY42-B* and some of the JA biosynthesis-related genes were under tight control to maintain a proper content of JA for normal growth in wheat. Before the initiation of leaf senescence, TaWRKY42-B exhibited a relatively high expression level at the juvenile stage for JA accumulation and JA-related developmental regulations, including preparation for the initiation of leaf senescence, which in turn activated *TaWRKY42-B* expression. As leaf senescence progressed, *TaWRKY42-B* and its targets, such as *TaLOX3*, underwent continuous suppression and cooperated with other senescence-related regulators for organized progression of leaf senescence. It is undeniable that JA content is almost continuously elevated with the progression of senescence, suggesting that accumulation of JA during leaf senescence is governed by other regulators. However, some detailed information on the underlying mechanism of the TaWRKY42-B-*TaLOX3* module and some in vivo experiments are needed to further validate our hypothesis.

Finally, based on our genetic data, we manifested that TaWRKY42-B involved leaf senescence regulation was partially conserved with AtWRKY53. Full-length TaWRKY42-B CDS driven by the AtWRKY53 promoter was able to rescue delayed leaf senescence in *atwrky53*. Previously, experimental data showed that AtWRKY53 was antagonistically regulated by JA and SA signaling in leaf senescence. In our study, we discovered that TaWRKY42-B modulating leaf senescence was also connected with JA, which further implied that TaWRKY42-B was functionally conserved with AtWRKY53 in age-dependent leaf senescence.

Briefly, we proposed a model of TaWRKY42-B-promoted leaf senescence (Fig. 9f). The transcription level of *TaWRKY42-B* was tightly controlled by the progression of leaf senescence and JA signaling. In non-senescent leaves, increased JA active *TaWRKY42-B* triggered the transcription of *TaLOX3* and subsequently, elevated JA biosynthesis for leaf senescence onset. However, at the senescence stage, TaWRKY42-B-*TaLOX3* was suppressed for organized leaf senescence progression. This feedback loop was likely to contribute to the proper timing of leaf senescence in wheat. Being functionally conserved with AtWRKY53, TaWRKY42-B was likewise involved in JA-related leaf senescence and altered the expression of some JA biosynthesis genes, such as *AtLOX3* and *TaLOX3*. Although more in vivo evidence is needed for comprehensive understanding of the functional role of TaWRKY42-B, we shed a light on the detailed information about the transcriptional regulation of leaf senescence in wheat.

## Conclusions

Collectively, by physiological and molecular analyses, we identified a novel WRKY-type promoter of leaf senescence, TaWRKY42-B, which mediated leaf senescence initiation by promoting JA biosynthesis and interacting with *AtLOX3* and *TaLOX3*. Our results provide a new clue for uncovering the mechanisms underlying wheat leaf senescence and a potential target gene for improving productivity and quality through modulating the leaf senescence process in wheat.

## Methods

### Plant materials and growth conditions

*Arabidopsis thaliana* ecotype ‘Col-0’ was obtained from the Arabidopsis Biological Resource Center (ABRC; http://abrc.osu.edu) and used for transformation. Arabidopsis mutant *atwrky53* (SALK_034157) was kindly provided by Prof. Ying Miao (Fujian Agriculture and Forestry University) and used for the complementation experiment. The *atwrky53* mutant had a T-DNA insertion within the second exon and genotyping of *atwrky53* in this study was described previously [41]. The delayed leaf senescence phenotype of *atwrky53* was also confirmed by authors through the measurement of senescence-related parameters. All Arabidopsis plants were grown in a growth chamber at Hebei Normal University. Surface-sterilized seeds were sown in 1/2MS medium containing 1% sucrose, 0.8% agar, and 1 × Murashige and Skoog salt, and treated at 4 °C for 48 h. Seven-day-old seedlings were transferred to the green house at 22 °C under long-day conditions (LD: 16 h light /8 h dark). Four-week-old plants grown in LD condition were treated with darkness and MeJA, and chlorophyll content and ion leakage were measured.

The bread wheat cultivar “ShiLuan 02–1” is widely cultivated for agricultural production in China. In this study, “ShiLuan 02–1” was kindly provided by Prof. Zhanjing Huang (Hebei Normal University) and used for BSMV-VIGS, and detection of *TaWRKY42-B* expression pattern. Seeds of “ShiLuan 02–1” were preserved in our lab and could also be obtained from the seed bank of the Institute of Genetics and Physiology, Hebei Academy of Agriculture and Forestry Sciences. Phenotypic characters and agronomic traits of “ShiLuan 02–1” were analyzed by the authors for the confirmation of the “ShiLuan 02–1” background. Wheat plants were grown under greenhouse conditions at Hebei Normal University and Hebei Academy of Agriculture and Forestry Sciences. Seven-day-old seedlings were used for infiltration. Five-week-old infiltrated plants were treated with darkness and the expression level of *TaWRY42-B* and *SAGs* was detected. For examining the expression level of *TaWRKY42-B* at four developmental leaf stages, wheat flag leaves were harvested from April to June; and the timing of collection was described in Arabidopsis based on the chlorophyll content, ion leakage rate, and expression level of *SAGs* [22]. The bread wheat cultivar “KeNong 199” was used to generate wheat protoplasts.

### Plasmid construction and plant transformation

For the overexpression of *TaWRKY42-B*, the full-length 879 bp CDS of *TaWRKY42-B* was amplified from the flag leaves of wheat and introduced to the pCAMBIA1300-GFP vector. The above vector was transferred into *Agrobacterium* strain GV3101 and transformed to Arabidopsis through the floral dip transformation method. TaWRKY42-B-GFP fusions in transgenic lines were detected by the western-blot assay with a GFP antibody.

To test transcriptional activity and subcellular localization, *TaWRKY42-B* was subcloned into pSAT-GAL4DB, pGBKT7, and PUC19 vectors.

For EMSA, full-length cDNA of *TaWRKY42-B* was generated and cloned into the pMAL-C2X vector and the construct was transformed into *E. coli* strain *Rosetta*.

To generate *TaWRKY42-B* BSMV-VIGS constructs, a 198 bp fragment of *TaWRKY42-B* was constructed into the pCaBS-γbLIC vector.

### Protoplast transformation and dual-luciferase reporter assay

Protoplasts were isolated from 7-day-old etiolated “KeNong 199” seedlings. pGreen0800-LUC was fused with the *TaLOX3* promoter and used as a reporter. *35S:TaWRKY42-B-GFP* was used as an effector and *35S:GFP* as a negative control. The plasmids pGreen0800-LUC and *35S:TaWRKY42-B-GFP* or *35S:GFP*, were co-transformed into wheat protoplasts and incubated for 18 h. The firefly and *Renilla* luciferase activity were quantified by the dual-luciferase assay kit (Promega, E1910).

### Ion leakage and chlorophyll content

The relative chlorophyll content of rosette leaves was detected with the SPAD502 Plus Chlorophyll Meter. For measurement of membrane ion leakage, 7th leaf of each plant was harvested and placed in deionized water under vacuum for 1 h. Conductivity of different background leaves were measured. Next, the conductivity of leaves which were boiled in deionized water for 15 min were measured and used as total conductivity. After subtracting the conductivity value of water, conductivity of leaves before and after boiling was used to generate the value of ion leakage rate. The ratio of conductivity of leaves before and after boiling was finally identified as ion leakage rate [19].

### qRT-PCR

Total RNA was extracted using the Trizol reagent (TAKARA, 9109). Total RNA (500 ng) was treated with 1 μL DNase I and used for preparing the first-strand cDNA. Synthesized cDNA was quantified by qRT-PCR. Real-time PCR was performed by mixing SYBR green (TAKARA, RR420A) with the ROX reference dye in ABI7300. Each sample was analyzed in three biological replicates. All the primers used in this study were listed in Additional file 7: Table S1. The expression levels of target genes were normalized to the expression level of the internal control, *TaACTIN* in wheat, and the expression levels were normalized to the expression level of the internal control, *AtUBC30* in Arabidopsis.

### RNA-Seq

Total RNA was isolated from Chinese Spring flag leaf blades at booting, heading, Anthesis and Grain-filling stage using TIANGEN® RNAprep Pure Plant Plus Kit (Polysaccharides&Polyphenolics-rich). Three biological replicates were employed. NEBNext® Ultra™ RNA Library Prep Kit for Illumina® (NEB, USA) was used to generate non-stranded sequencing libraries according to instructions. TruSeq PE Cluster Kit v3-cBot-HS (Illumina) was used to cluster the index-coded samples by the cBot Cluster Generation System according to the manufacturer’s recommendations. Next, Illumina Novaseq6000 platform was used to perform the sequencing of the libraries, therefore the reads with 150 bp paired-end were generated. The overall sequencing quality of the reads in each sample was evaluated and controlled with the software Fastp by using default parameters [70]. The remaining reads were aligned to the wheat reference genome IWGSCv1.0 (http://www.wheatgenome.org/) using HISAT2 v2.1 (https://daehwankimlab.github.io/hisat2/) with the default parameters, and only the uniquely mapped reads were retained for the following analysis (Additional file 8: Table S2). The gene expression quantification was done by HTseq [71, 72]. The R package DESeq2 was used to perform differential expression analysis between each two stages, and only the genes with an absolute value of log_2_ (fold change) ≥1 and *P* value < 0.05 were considered as DEGs.

The RNA sequencing data have been deposited into NCBI Sequence Read Archive (https://www.ncbi.nlm.nih.gov/bioproject/PRJNA656068) under the accession no. PRJNA656068 and CNSA (https://db.cngb.org/search/project/CNP0001003) with the accession no. CNP0001003.

### Phylogenetic analysis and gene expression

The phylogenetic tree was constructed by MEGA X using the maximum likelihood method with WAG+F model [73] and outputted as a Newick file, which was uploaded to the Interactive Tree Of Life (https://itol.embl.de/), an online tool for tree design, to create the figure. The gene expression levels were based on FPKM values from RNA-Seq data and standardized with log2(FPKM+ 1). The heatmap was constructed with TBtools [74].

### Dark-induced leaf senescence

For dark-induced leaf senescence, One-month-old wheat leaves and 6th and 7th rosette leaves of 4-week-old Arabidopsis plants were placed into dishes with 10 mL deionized water. The samples were kept in the dark for 5 or 6 days, and then the chlorophyll content and the ion leakage were measured.

For JA-induced leaf senescence, the 6th and 7th leaves were treated with deionized water or 100 μM MeJA solution and the samples were kept in the dark at 22 °C for 4 or 5 days. Then the chlorophyll content and ion leakage were measured.

### DAB staining

Deionized water was adjusted to a pH of 3 with HCl, 1% DAB was added until completely dissolved, and then, the pH was adjusted to 5.7 with KOH. The detached leaves of Arabidopsis and wheat were immersed in 1% DAB solution and under a vacuum condition for 15 min. Next, above leaves were incubated in darkness for another 4–6 h at room temperature. Samples were placed in 75% alcohol until discolored.

### BSMV

The target fragment of *TaWRKY42-B* was cloned into the pCaBS-γbLIC plasmid by the LIC strategy and equal amounts of bacteria harboring pCaBS-α, pCaBS-β, and pCaBS-γbLIC were mixed and infiltrated into *N. benthamiana* leaves. The solution contained 10 mM MgCl_2_, 10 mM MES, and 0.1 M Acetyleugenone (pH = 5.2). Seven days after infiltration, the infected local leaves were harvest for the sap and the sap was mechanically inoculated onto wheat at the two-leaf stage. The presence of the vector BSMV::TaWRKY42-B_198_ in *N. benthamiana* could be detected after 5 days of infection, which indicated successful infection. In addition, BSMV::TaPDS, as the positive control, and the empty (pCaBS-α, pCaBS-β and pCaBS-γbLIC) as negative controls.

### JA quantification

Three-week-old and 5-week-old *TaWRKY42-B-OE* and Col-0 Arabidopsis plants for JA analysis were processed and purified as previously described [75]. Detached leaves (200 mg) of *TaWRKY42-B-OE* and Col-0 plants were homogenized and immersed in methanol for 24 h. After centrifugation of all samples, purification was conducted by using the Oasis Max solid phase extract cartridge. JA content of all samples were measured by UPLC system (Waters) and QTRAP 5500 system (AB SCIEX). Three biological replicates were performed and ^2^H_5_-JA was used as the internal reference standard.

### EMSA

For EMSA, the TaWRKY42-B protein fused with MBP was expressed in *E. coli* (Rosetta) and purified with Amylose Resin (0812S, New England Biolabs, USA). Expression of the TaWRKY42-B-MBP fusion protein was induced in 200 mL cultures of transformed bacteria by the addition of IPTG to a final concentration of 0.5 mM; and cultures were incubated at 25 °C for 6 h. Biotin-labeled DNA probes are listed in Additional file 7: Table S1. Unlabeled competitor probes were added at 100-fold and 200-fold molar excess. EMSA was performed by using the Chemiluminescent Nucleic Acid Detection Module (Thermo Scientific, 89,880). A total reaction volume of 10 μL contained 1 μL binding buffer, 0.5 μL glycerol, 0.5 μL poly-dIdC, 1 μL biotin-probe, 0.5 μL 1 M KCl, and 400 ng purified fusion protein. Biotin-labeled DNA was detected using the Stable Peroxide Solution (Thermo Scientific, 89,880).

## Supplementary information

**Additional file 1: Figure S1.** Sequence alignment of *TaWRKY42-B*, *TaWRKY42-A*, and *TaWRKY42-D.* (a) Cluster analysis among WRKY subgroups and TaWRKY42-B. (b) Alignment of nucleotide sequences of *TaWRKY42-B, TaWRKY42-A*, and *TaWRKY42-D.* (c) Alignment of TaWRKY42-B*,* TaWRKY42-A, and TaWRKY42-D protein sequences in DNAMAN.

**Additional file 2: Figure S2.** The target fragment of *TaWRKY42-B* for BSMV-VIGS. (a) Sequence of the target fragment of *TaWRKY42-B* in BSMV-VIGS. (b) Detection of BSMV::TaWRKY42-B_198_ vector in infiltrated *N. benthamiana*. BSMV::00 was used as a negative control and BSMV::TaWRKY42-B_198_ plasmid served as a positive control (PC). Lane 1 and 2 showed the PCR produces of BSMV::TaWRKY42-B198 vector in two infiltrated leaves, respectively.

**Additional file 3: Figure S3.** TaWRKY42-B possesses transcriptional activity in yeast. (a) Yeast strains containing the indicated vectors were grown on SD/−Trp-His-Leu medium. The strain contained the pGBKT7 vector as the negative control. *TaNAC6* and *TaWRKY5-D* were fused to pGBKT7 as the positive control. (b) The above yeast colonies were analyzed by X-gal staining.

**Additional file 4: Figure S4.** Promoter analysis of *TaWRKY42-B* in PlantCARE. The localization of *cis*-elements in *TaWRKY42-B* promoter region. The CGTCA-motif was involved in MeJA responsiveness, TCA-element was involved in SA responsiveness, and ABRE was involved in ABA responsiveness.

**Additional file 5: Figure S5.** Overexpression of *TaWRKY42-B* promotes dark-induced leaf senescence. (a) Detached leaves of 4-week-old Col-0 and *TaWRKY42-B-*overexpressing plants were treated with darkness for 6 days. Chlorophyll content (b) and ion leakage rate (c) of detached leaves before and after dark treatment as shown in (a). (Error bars indicate SD. Asterisks indicate significant differences in SPAD and ion leakage between *TaWRKY42-B-OE* lines and Col-0. Student’s *t-*test, *P < 0.05, **P < 0.01).

**Additional file 6: Figure S6.** TaWRKY42-B cannot directly bind to the promotor of *AtLOX1*. (a) Diagram of probes against *AtLOX1* promoter region for EMSA. (b) The interaction between TaWRKY42-B and promoter region of *AtLOX1* was analyzed by EMSA. TaWRKY42-B-MBP fusion protein mixed with labeled probes and 100× or 200× unlabeled probes served as competitors. The presence (+) or absence (−) of specific probes existence or not. Numbers above the top of the bands indicate the relative binding strength of the TaWRKY42-B-MBP fusion protein and labeled probes after normalization to the control group.

**Additional file 7: Table S1.** Primers and probes used in this study.

**Additional file 8: Table S2.** Number of reads in each library.

## Data Availability

RNA-seq data has been deposited into CNSA (https://db.cngb.org/search/project/CNP0001003) with accession no. CNP0001003 and NCBI Sequence Read Archive (https://www.ncbi.nlm.nih.gov/bioproject/PRJNA656068) under the accession no. PRJNA656068. All the other experimental data about in this study are presented in this article and its supplementary information files.

## Abbreviations

ABA: Abscisic acid
AUXs: Auxins
BRs: Brassinosteroids
BSMV: *Barley stripe mosaic virus*
CKs: Cytokinins
DAB: 3,3-diaminobenzidine
DAT: Day after treatment
EMSA: Electrophoretic mobility shift assay
GA: Gibberellic acid
JA: Jasmonic acid
LUC: Firefly luciferase
MeJA: Methyl jasmonate
SA: Salicylic acid
SAGs: Senescence-associated genes
SLs: Strigolactones
REN: *Renilla* luciferase
TF: Transcription factor
VIGS: Virus-induced gene silencing
WT: Wild type
