# Supplementary material for: A WRKY transcription factor, TaWRKY42-B, facilitates initiation of leaf senescence by promoting jasmonic acid biosynthesis
Source: BMC Plant Biol. 2020 Sep 29;20:444. doi: 10.1186/s12870-020-02650-7 (PMC7526184; doi:10.1186/s12870-020-02650-7)
Supplement: Supplementary file 1 — Additional file 1: Figure S1. Sequence alignment of TaWRKY42-B, TaWRKY42-A, and TaWRKY42-D. (a) Cluster analysis among WRKY subgroups and TaWRKY42-B. (b) Alignment of nucleotide sequences of TaWRKY42-B, TaWRKY42-A, and TaWRKY42-D. (c) Alignment of TaWRKY42-B, TaWRKY42-A, and TaWRKY42-D protein sequences in DNAMAN. [file 12870_2020_2650_MOESM1_ESM.pptx]

## Slide 1
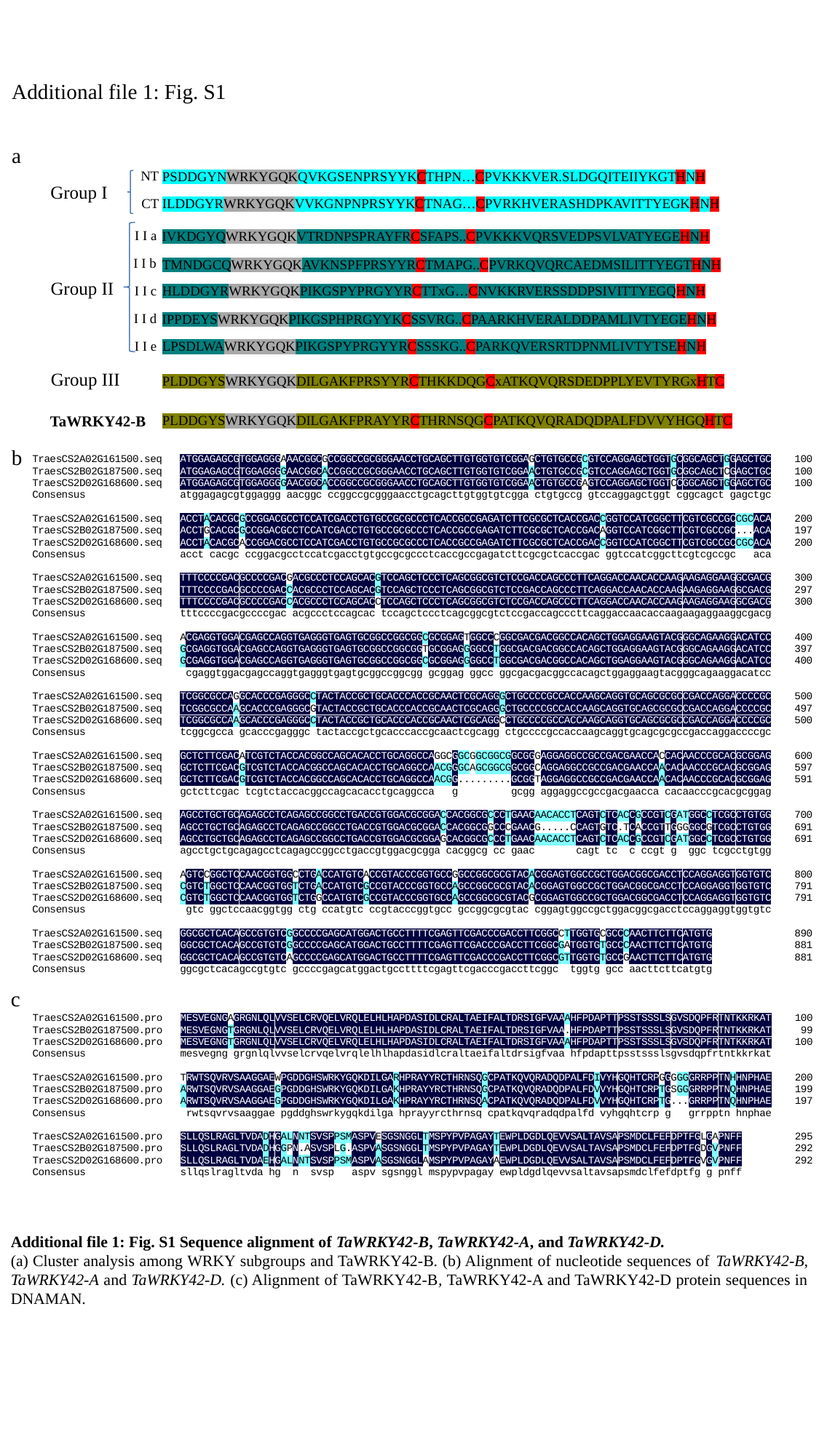

Additional file 1: Fig. S1
a
NT
Group I
CT
I I a
I I b
Group II
I I c
I I d
I I e
Group III
TaWRKY42-B
PSDDGYNWRKYGQKQVKGSENPRSYYKCTHPN…CPVKKKVER.SLDGQITEIIYKGTHNH
ILDDGYRWRKYGQKVVKGNPNPRSYYKCTNAG…CPVRKHVERASHDPKAVITTYEGKHNH
IVKDGYQWRKYGQKVTRDNPSPRAYFRCSFAPS..CPVKKKVQRSVEDPSVLVATYEGEHNH
TMNDGCQWRKYGQKAVKNSPFPRSYYRCTMAPG..CPVRKQVQRCAEDMSILITTYEGTHNH
HLDDGYRWRKYGQKPIKGSPYPRGYYRCTTxG…CNVKKRVERSSDDPSIVITTYEGQHNH
IPPDEYSWRKYGQKPIKGSPHPRGYYKCSSVRG..CPAARKHVERALDDPAMLIVTYEGEHNH
LPSDLWAWRKYGQKPIKGSPYPRGYYRCSSSKG..CPARKQVERSRTDPNMLIVTYTSEHNH
PLDDGYSWRKYGQKDILGAKFPRSYYRCTHKKDQGCxATKQVQRSDEDPPLYEVTYRGxHTC
PLDDGYSWRKYGQKDILGAKFPRAYYRCTHRNSQGCPATKQVQRADQDPALFDVVYHGQHTC
b
c
Additional file 1: Fig. S1 Sequence alignment of TaWRKY42-B, TaWRKY42-A, and TaWRKY42-D.
(a) Cluster analysis among WRKY subgroups and TaWRKY42-B. (b) Alignment of nucleotide sequences of TaWRKY42-B, TaWRKY42-A and TaWRKY42-D. (c) Alignment of TaWRKY42-B, TaWRKY42-A and TaWRKY42-D protein sequences in DNAMAN.
