# Supplementary material for: A WRKY transcription factor, TaWRKY42-B, facilitates initiation of leaf senescence by promoting jasmonic acid biosynthesis
Source: BMC Plant Biol. 2020 Sep 29;20:444. doi: 10.1186/s12870-020-02650-7 (PMC7526184; doi:10.1186/s12870-020-02650-7)
Supplement: Supplementary file 2 — Additional file 2: Figure S2. The target fragment of TaWRKY42-B for BSMV-VIGS. (a) Sequence of the target fragment of TaWRKY42-B in BSMV-VIGS. (b) Detection of BSMV::TaWRKY42-B198 vector in infiltrated N. benthamiana. BSMV::00 was used as a negative control and BSMV::TaWRKY42-B198 plasmid served as a positive control (PC). Lane 1 and 2 showed the PCR produces of BSMV::TaWRKY42-B198 vector in two infiltrated leaves, respectively. [file 12870_2020_2650_MOESM2_ESM.pptx]

## Slide 1
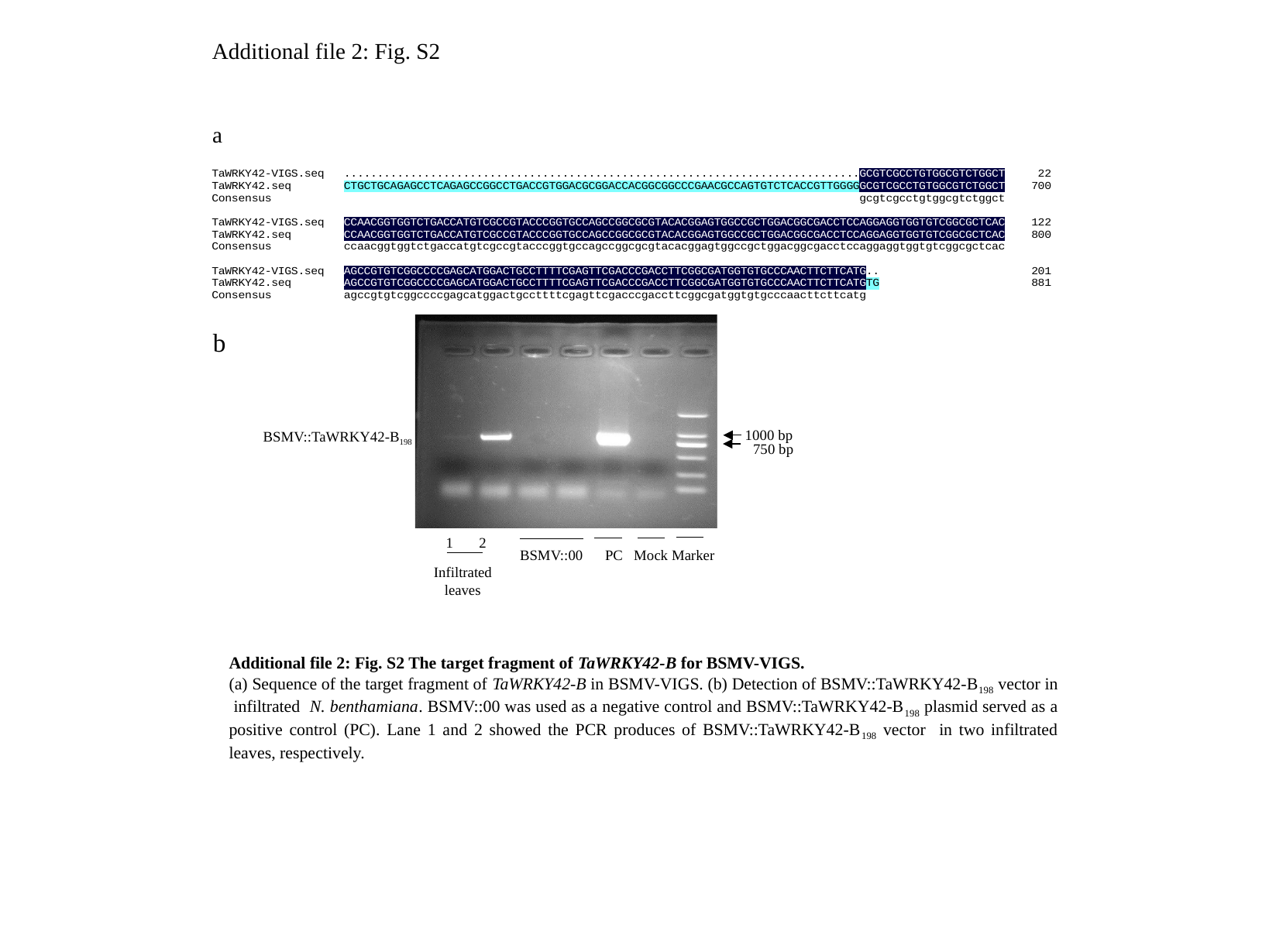

Additional file 2: Fig. S2
a
b
1000 bp
BSMV::TaWRKY42-B198
750 bp
1 2
 BSMV::00 PC Mock Marker
Infiltrated
leaves
Additional file 2: Fig. S2 The target fragment of TaWRKY42-B for BSMV-VIGS.
(a) Sequence of the target fragment of TaWRKY42-B in BSMV-VIGS. (b) Detection of BSMV::TaWRKY42-B198 vector in infiltrated N. benthamiana. BSMV::00 was used as a negative control and BSMV::TaWRKY42-B198 plasmid served as a positive control (PC). Lane 1 and 2 showed the PCR produces of BSMV::TaWRKY42-B198 vector in two infiltrated leaves, respectively.
