# Supplementary material for: A WRKY transcription factor, TaWRKY42-B, facilitates initiation of leaf senescence by promoting jasmonic acid biosynthesis
Source: BMC Plant Biol. 2020 Sep 29;20:444. doi: 10.1186/s12870-020-02650-7 (PMC7526184; doi:10.1186/s12870-020-02650-7)
Supplement: Supplementary file 3 — Additional file 3: Figure S3. TaWRKY42-B possesses transcriptional activity in yeast. (a) Yeast strains containing the indicated vectors were grown on SD/−Trp-His-Leu medium. The strain contained the pGBKT7 vector as the negative control. TaNAC6 and TaWRKY5-D were fused to pGBKT7 as the positive control. (b) The above yeast colonies were analyzed by X-gal staining. [file 12870_2020_2650_MOESM3_ESM.pptx]

## Slide 1
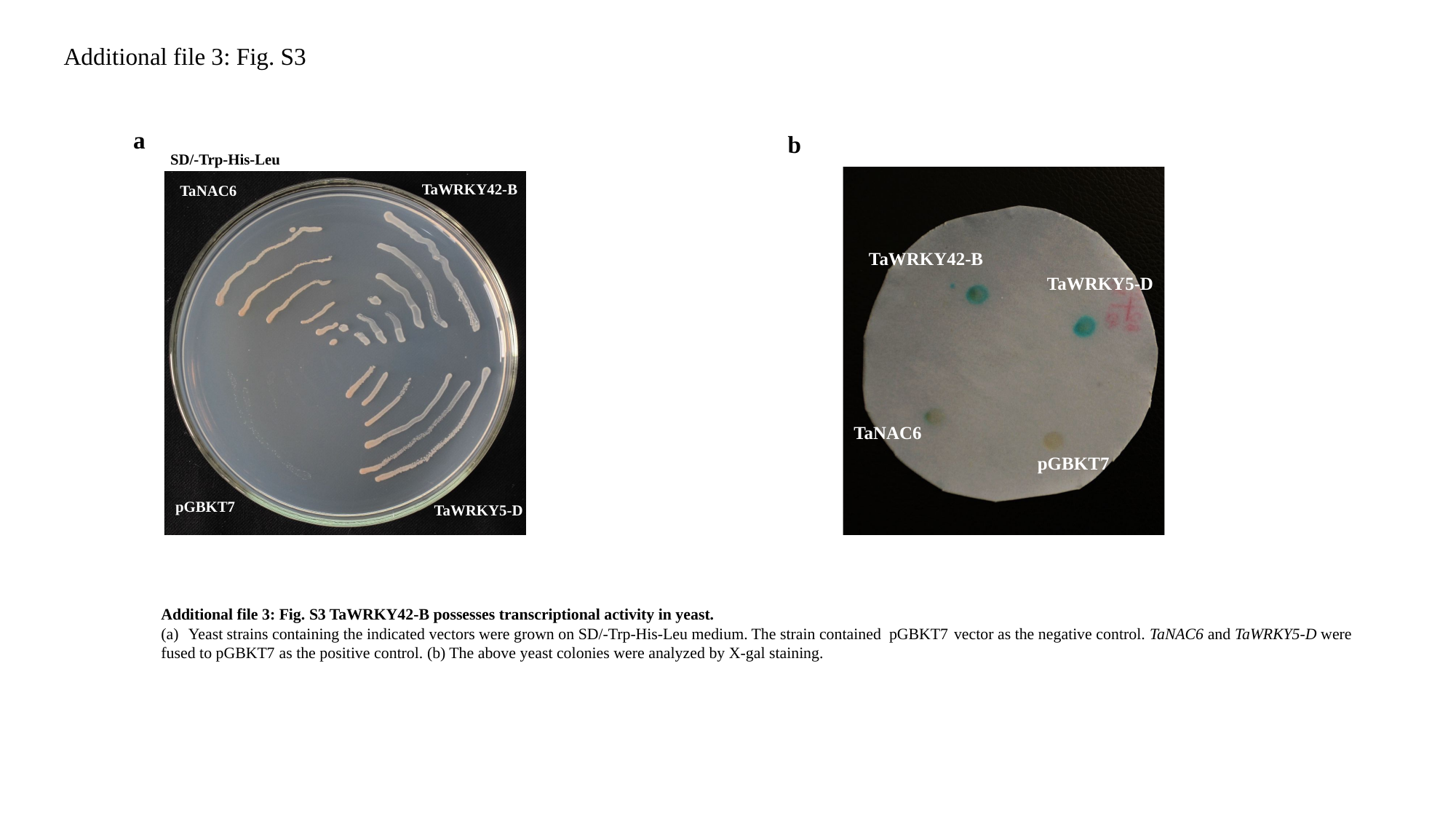

Additional file 3: Fig. S3
a
b
SD/-Trp-His-Leu
TaWRKY42-B
 TaNAC6
TaWRKY42-B
TaWRKY5-D
TaWRKY5-D
TaNAC6
pGBKT7
pGBKT7
TaWRKY5-D
Additional file 3: Fig. S3 TaWRKY42-B possesses transcriptional activity in yeast.
Yeast strains containing the indicated vectors were grown on SD/-Trp-His-Leu medium. The strain contained pGBKT7 vector as the negative control. TaNAC6 and TaWRKY5-D were
fused to pGBKT7 as the positive control. (b) The above yeast colonies were analyzed by X-gal staining.
