# Supplementary material for: A WRKY transcription factor, TaWRKY42-B, facilitates initiation of leaf senescence by promoting jasmonic acid biosynthesis
Source: BMC Plant Biol. 2020 Sep 29;20:444. doi: 10.1186/s12870-020-02650-7 (PMC7526184; doi:10.1186/s12870-020-02650-7)
Supplement: Supplementary file 4 — Additional file 4: Figure S4. Promoter analysis of TaWRKY42-B in PlantCARE. The localization of cis-elements in TaWRKY42-B promoter region. The CGTCA-motif was involved in MeJA responsiveness, TCA-element was involved in SA responsiveness, and ABRE was involved in ABA responsiveness. [file 12870_2020_2650_MOESM4_ESM.pptx]

## Slide 1
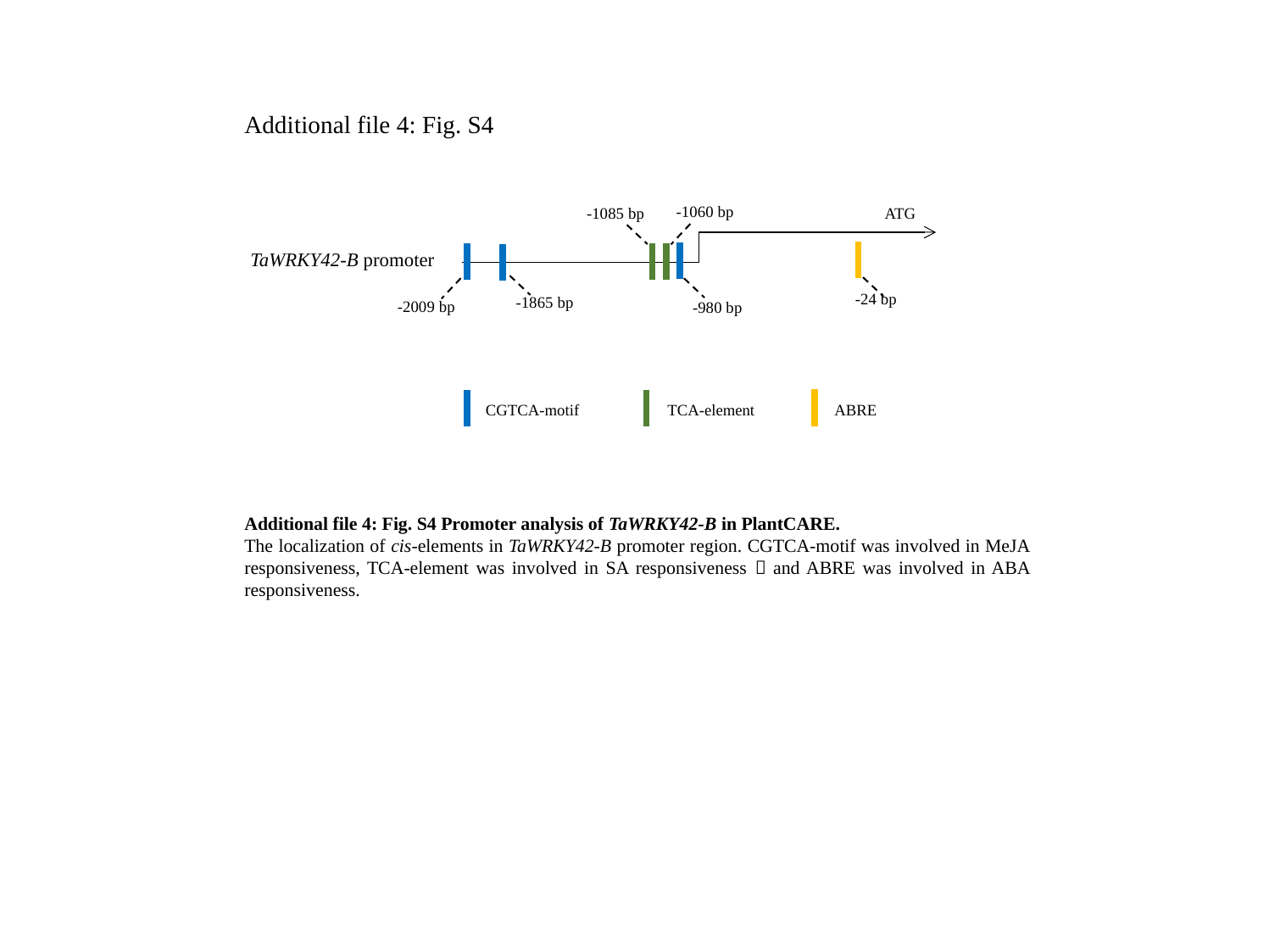

Additional file 4: Fig. S4
-1060 bp
ATG
-1085 bp
-24 bp
-1865 bp
-2009 bp
-980 bp
CGTCA-motif TCA-element ABRE
TaWRKY42-B promoter
Additional file 4: Fig. S4 Promoter analysis of TaWRKY42-B in PlantCARE.
The localization of cis-elements in TaWRKY42-B promoter region. CGTCA-motif was involved in MeJA responsiveness, TCA-element was involved in SA responsiveness，and ABRE was involved in ABA responsiveness.
