# Supplementary material for: A WRKY transcription factor, TaWRKY42-B, facilitates initiation of leaf senescence by promoting jasmonic acid biosynthesis
Source: BMC Plant Biol. 2020 Sep 29;20:444. doi: 10.1186/s12870-020-02650-7 (PMC7526184; doi:10.1186/s12870-020-02650-7)
Supplement: Supplementary file 5 — Additional file 5: Figure S5. Overexpression of TaWRKY42-B promotes dark-induced leaf senescence. (a) Detached leaves of 4-week-old Col-0 and TaWRKY42-B-overexpressing plants were treated with darkness for 6 days. Chlorophyll content (b) and ion leakage rate (c) of detached leaves before and after dark treatment as shown in (a). (Error bars indicate SD. Asterisks indicate significant differences in SPAD and ion leakage between TaWRKY42-B-OE lines and Col-0. Student’s t-test, *P < 0.05, **P < 0.01). [file 12870_2020_2650_MOESM5_ESM.pptx]

## Slide 1
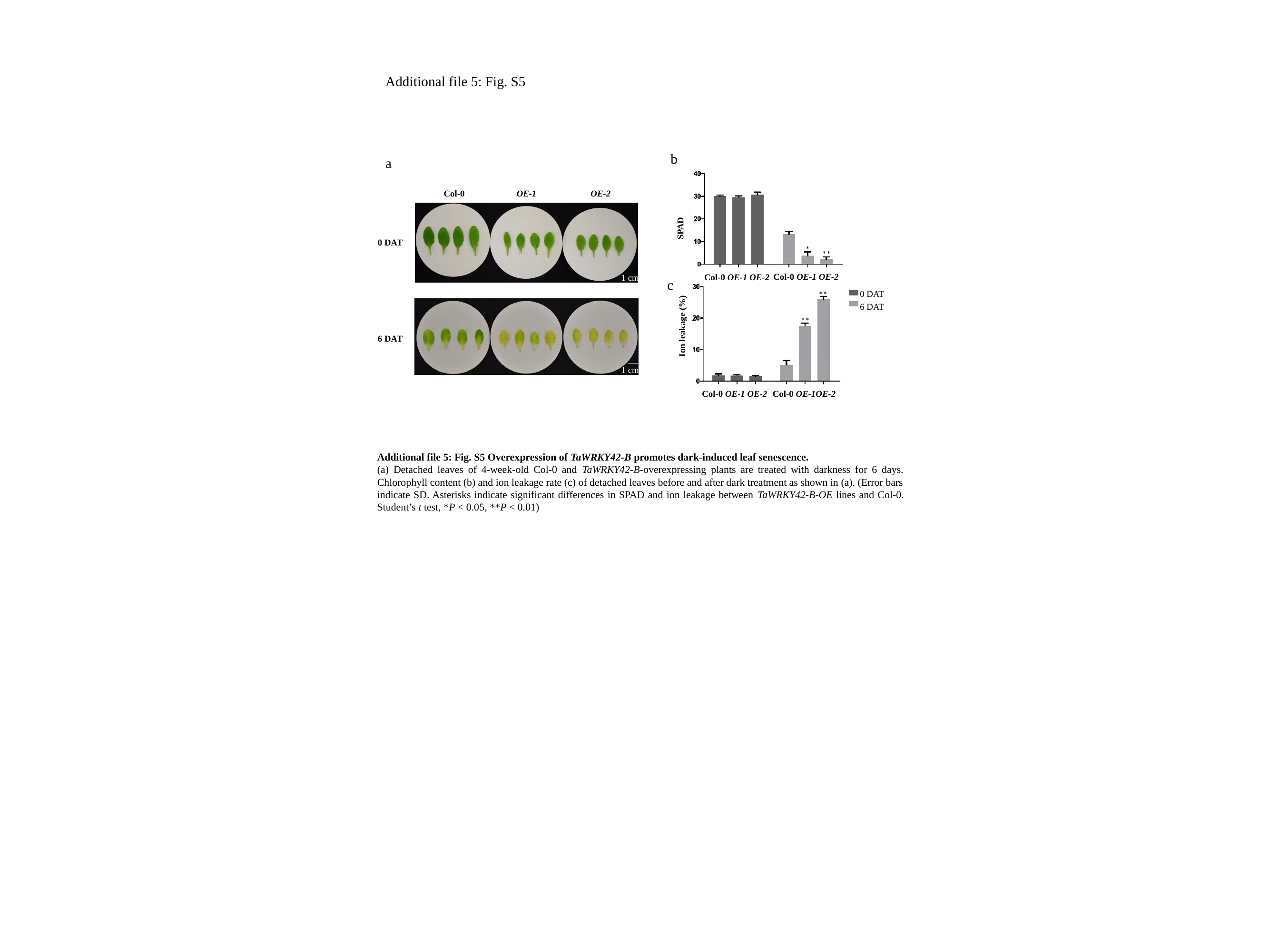

Additional file 5: Fig. S5
b
a
SPAD
*
**
Col-0 OE-1 OE-2
Col-0 OE-1 OE-2
 Col-0 OE-1 OE-2
0 DAT
6 DAT
1 cm
c
0 DAT
6 DAT
**
Ion leakage (%)
**
Col-0 OE-1 OE-2
Col-0 OE-1OE-2
1 cm
Additional file 5: Fig. S5 Overexpression of TaWRKY42-B promotes dark-induced leaf senescence.
(a) Detached leaves of 4-week-old Col-0 and TaWRKY42-B-overexpressing plants are treated with darkness for 6 days. Chlorophyll content (b) and ion leakage rate (c) of detached leaves before and after dark treatment as shown in (a). (Error bars indicate SD. Asterisks indicate significant differences in SPAD and ion leakage between TaWRKY42-B-OE lines and Col-0. Student’s t test, *P < 0.05, **P < 0.01)
