# Supplementary material for: A WRKY transcription factor, TaWRKY42-B, facilitates initiation of leaf senescence by promoting jasmonic acid biosynthesis
Source: BMC Plant Biol. 2020 Sep 29;20:444. doi: 10.1186/s12870-020-02650-7 (PMC7526184; doi:10.1186/s12870-020-02650-7)
Supplement: Supplementary file 6 — Additional file 6: Figure S6. TaWRKY42-B cannot directly bind to the promotor of AtLOX1. (a) Diagram of probes against AtLOX1 promoter region for EMSA. (b) The interaction between TaWRKY42-B and promoter region of AtLOX1 was analyzed by EMSA. TaWRKY42-B-MBP fusion protein mixed with labeled probes and 100× or 200× unlabeled probes served as competitors. The presence (+) or absence (−) of specific probes existence or not. Numbers above the top of the bands indicate the relative binding strength of the TaWRKY42-B-MBP fusion protein and labeled probes after normalization to the control group. [file 12870_2020_2650_MOESM6_ESM.pptx]

## Slide 1
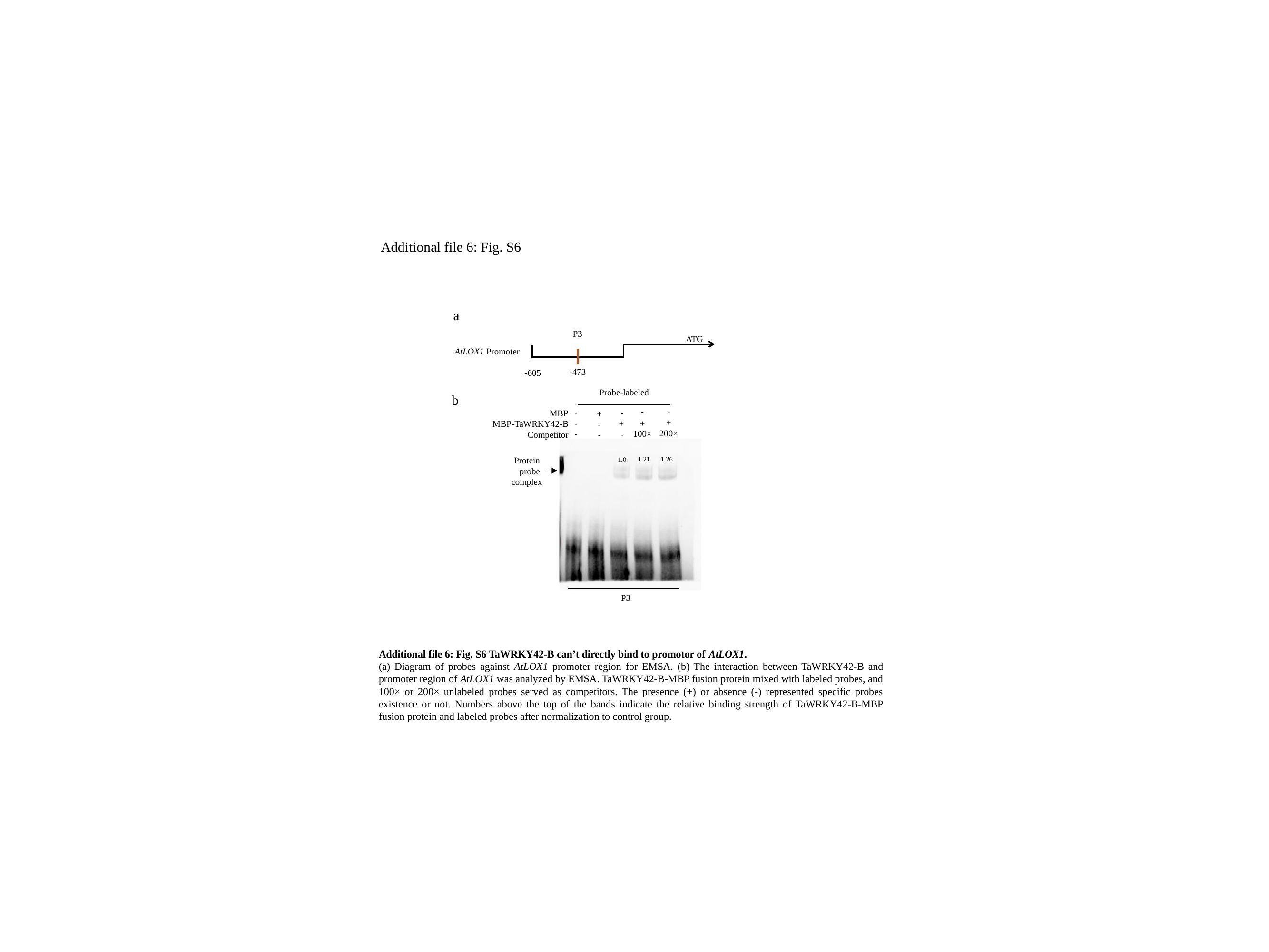

Additional file 6: Fig. S6
a
P3
ATG
AtLOX1 Promoter
-473
-605
Probe-labeled
b
-
+
200×
-
+
100×
-
-
-
MBP
MBP-TaWRKY42-B
Competitor
-
-
+
-
-
+
1.21
Protein
probe
complex
1.26
1.0
P3
Additional file 6: Fig. S6 TaWRKY42-B can’t directly bind to promotor of AtLOX1.
(a) Diagram of probes against AtLOX1 promoter region for EMSA. (b) The interaction between TaWRKY42-B and promoter region of AtLOX1 was analyzed by EMSA. TaWRKY42-B-MBP fusion protein mixed with labeled probes, and 100× or 200× unlabeled probes served as competitors. The presence (+) or absence (-) represented specific probes existence or not. Numbers above the top of the bands indicate the relative binding strength of TaWRKY42-B-MBP fusion protein and labeled probes after normalization to control group.
